# Molecular surveillance for operationally relevant genetic polymorphisms in *Plasmodium falciparum* in Southern Chad, 2016–2017

**DOI:** 10.1186/s12936-022-04095-9

**Published:** 2022-03-12

**Authors:** Sukanta Das, Clément Kérah-Hinzoumbé, Moundiné Kebféné, Suttipat Srisutham, Tog-Yeum Nagorngar, Naowarat Saralamba, Ranitha Vongpromek, Teeradet Khomvarn, Carol H. Sibley, Philippe J. Guérin, Mallika Imwong, Mehul Dhorda

**Affiliations:** 1 Department of Molecular Tropical Medicine, Faculty of Tropical Medicine, Mahidol University, Bangkok, Thailand; 2 Programme National de Lutte Contre Le Paludisme, N’Djamena, Republic of Chad; 3 ExxonMobil, N’Djamena, Republic of Chad; 4 WorldWide Antimalarial Resistance Network – Asia-Pacific Regional Centre, Bangkok, Thailand; 5 Mahidol-Oxford Tropical Medicine Research Unit, Faculty of Tropical Medicine, Mahidol University, Bangkok, Thailand; 6 WorldWide Antimalarial Resistance Network, Oxford, UK; 7 Department of Genome Sciences, University of Washington, Seattle, WA USA; 8 Centre for Tropical Medicine and Global Health, Nuffield Department of Medicine, University of Oxford, Oxford, UK; 9 Department of Clinical Microscopy, Faculty of Allied Health Sciences, Chulalongkorn University, Bangkok, Thailand

## Abstract

**Background:**

Resistance to anti-malarials is a serious threat to the efforts to control and eliminate malaria. Surveillance based on simple field protocols with centralized testing to detect molecular markers associated with anti-malarial drug resistance can be used to identify locations where further investigations are needed.

**Methods:**

Dried blood spots were collected from 398 patients (age range 5–59 years, 99% male) with *Plasmodium falciparum* infections detected using rapid diagnostic tests over two rounds of sample collection conducted in 2016 and 2017 in Komé, South-West Chad. Specimens were genotyped using amplicon sequencing or qPCR for validated markers of anti-malarial resistance including partner drugs used in artemisinin-based combination therapy (ACT).

**Results:**

No mutations in the *pfk13* gene known to be associated with artemisinin resistance were found but a high proportion of parasites carried other mutations, specifically K189T (190/349, 54.4%, 95%CI 49.0–59.8%). Of 331 specimens successfully genotyped for *pfmdr1* and *pfcrt*, 52% (95%CI 46.4–57.5%) carried the NFD-K haplotype, known to be associated with reduced susceptibility to lumefantrine. Only 20 of 336 (6.0%, 95%CI 3.7–9.0%) had parasites with the *pfmdr1*-N86Y polymorphism associated with increased treatment failures with amodiaquine. Nearly all parasites carried at least one mutation in *pfdhfr* and/or *pfdhps* genes but ‘sextuple’ mutations in pfdhfr—pfdhps including pfdhps -A581G were rare (8/336 overall, 2.4%, 95%CI 1.2–4.6%). Only one specimen containing parasites with pfmdr1 gene amplification was detected.

**Conclusions:**

These results provide information on the likely high efficacy of artemisinin-based combinations commonly used in Chad, but suggest decreasing levels of sensitivity to lumefantrine and high levels of resistance to sulfadoxine-pyrimethamine used for seasonal malaria chemoprevention and intermittent preventive therapy in pregnancy. A majority of parasites had mutations in the *pfk13* gene, none of which are known to be associated with artemisinin resistance. A therapeutic efficacy study needs to be conducted to confirm the efficacy of artemether-lumefantrine.

**Supplementary Information:**

The online version contains supplementary material available at 10.1186/s12936-022-04095-9.

## Background

Resistance to anti-malarial drugs threatens recent gains in malaria control efforts and again poses a significant public health problem. The emergence in Southeast Asia and the subsequent global spread of chloroquine-resistant malaria was a major factor contributing to the failure of the first global malaria eradication campaign in the mid-twentieth century [1]. The widespread implementation of highly effective artemisinin-based combination therapy (ACT) for malaria has contributed to significant gains in global control and elimination efforts. Malaria elimination is now back on the agenda, 40 years after the first global malaria eradication campaign was abandoned [2]. However, the gains seen in the past decade are again at risk as parasite resistance to artemisinin compounds has been confirmed in Southeast Asia and more recently in Rwanda [3–9]. Further, mutations associated with artemisinin resistance have also been observed in New Guinea, Tanzania and Uganda [10–12]. Given the lack of immediately available new drugs and widely available efficacious and cheap vaccines, it is critical to prolong the usable life of currently available anti-malarial drugs by judicious implementation of treatment strategies.

In order to ensure that anti-malarial treatments with the greatest likely therapeutic efficacy are used, periodic assessments of drug resistance need to be performed in malaria endemic regions. The gold standard for such assessments is in vivo clinical trials of drug efficacy or Therapeutic Efficacy Studies (TES) per the terminology adopted by the World Health Organization (WHO). Such trials are relatively labour intensive, expensive and are often conducted at established sentinel sites where drug resistance may only be observed after it is already well established [13]. Areas of low transmission also tend to be the regions where anti-malarial drug resistance is selected, but sufficiently rapid enrolment of patients into drug efficacy studies can be hard to achieve. Supplementing clinical efficacy data with assessment of molecular markers for drug resistance can thus be valuable in monitoring for drug resistance. A large body of published work describes the advantages of molecular markers over standard in vivo and in vitro methods for monitoring resistance [14, 15], the validation of molecular markers as tools for surveillance [16–21], and the usefulness as well as the limitations of these markers to guide treatment policies [22].

The WHO recommends that anti-malarial treatments should be only be administered in cases where the diagnosis of malaria has been confirmed with a laboratory test [23]. Microscopy remains one of the most commonly performed diagnostic tests for malaria but is being replaced by Rapid Diagnostic Tests (RDTs) in most endemic regions. Some of the earliest and most widely used RDTs are based on the detection of *Plasmodium falciparum* histidine-rich proteins (PfHRP2) encoded by the *pfhrp2/3* genes. RDTs detecting other parasite antigens (lactate dehydrogenase, aldolase) exist, some of which can be less sensitive and/or more expensive than those based on detection of PfHRP2. There have been reports of *P. falciparum* ‘diagnosis-resistant’ parasites carrying partial or complete deletions in *pfhrp2/3* genes which produce little or no PfHRP2, leading to false negative results from RDTs [24]. Monitoring for the emergence of such mutations, and changing diagnostic practices if such emergence is confirmed, would help to ensure that the most accurate diagnostic tests are used to identify cases requiring anti-malarial treatment.

The report presented here describes a molecular surveillance study performed in the Republic of Chad, where an estimated 3 million cases of malaria occur every year [25]. The first-line treatment in Chad for uncomplicated *P. falciparum* malaria is artemisinin-based combination therapy (ACT), using either artemether-lumefantrine (AL) or artesunate-amodiaquine (ASAQ). Malaria prophylaxis as intermittent preventive treatment in pregnancy (IPTp) with sulfadoxine-pyrimethamine (SP) and seasonal malaria chemoprevention (SMC) with SP-AQ is provided to pregnant women and children < 5 years old, respectively. Only two therapeutic efficacy studies of ASAQ have been performed in the country and no monitoring has been performed for parasites for *pfhrp2/3* deletions [25]. The primary objective of the study was to measure the prevalence of parasites carrying mutations relevant to malaria control efforts in the country, i.e., those associated with reduced susceptibility to anti-malarial drugs and with increased rates of false negative results from RDTs.

## Methods

### Study site and population

This cross-sectional observational study was conducted in Komé in the southern part of the Republic of Chad at a private clinic serving employees, contractors and visitors of a local petroleum extraction site. The study was performed in two rounds during successive peak malaria transmission seasons. The sample size of approximately 200 participants per sample collection round was calculated assuming a prevalence of a marker of 5% with a desired precision (95% confidence interval) of ± 3%. Patients aged 6 months to 75 years who provided written informed consent (from parents or guardians of patients < 18 years) and experiencing symptoms of malaria (including but not limited to headache, body aches, fever, chills, and weakness) with no signs of severe malaria were eligible to participate.

### Sample collection and processing

Blood from those who provided written consent was used to prepare a dried blood spot (DBS) on filter paper at the same time when a malaria RDT was performed. In the second round of testing, an additional PfLDH RDT (CareStart Malaria pLDH Pf/Pan, Cat No G0121) was performed only in cases where the initial PfHRP2 test (SD Bioline Malaria Ag Pf/Pan, Cat No 05FK60; used in both rounds) was negative. If the second RDT was positive, the sample was flagged for additional testing to detect *pfhrp2/3* deletions. All patients with a confirmed malaria infection received anti-malarial treatment free of charge per the national treatment guideline.

Only DBS from persons with *P. falciparum* infection confirmed with a positive RDT result were retained for further molecular testing. Each DBS was assigned a unique identification number and stored in a separate resealable plastic bag with silica gel desiccant until DNA extraction. The unique identifier was recorded along with the date of sample collection, the age and sex of the participants and, in the second round, the RDT test results. The collected samples along with the corresponding logs were shipped to the Asia–Pacific Regional Centre of the WorldWide Antimalarial Resistance Network in Bangkok, Thailand. DNA extraction from the DBS was performed using the semi-automated QIASymphony® platform and Qiagen DNA Mini Kits.

### Genotyping for molecular markers of resistance and *pfhrp2/3* deletions

Samples were genotyped at the Molecular Tropical Medicine Laboratory, Faculty of Tropical Medicine, Mahidol University in Bangkok using established protocols to detect molecular markers of anti-malarial drug resistance and *pfhrp2/3* deletions (see Additional file 1 for further details). In brief, DNA extracted from the DBS was used as the template for amplification by polymerase chain reaction (PCR). To detect nucleotide sequence polymorphisms, PCR products were cleaned using the Favoprep™ PCR Purification kit per manufacturer’s instructions and sent to a commercial service for Sanger sequencing (Macrogen Inc, South Korea). Alignment of sequences received from the service provider was performed using Clustal (http://www.clustal.org) using reference sequences retrieved from PlasmoDB (www.plasmodb.org) and NCBI® Genbank® (https://www.ncbi.nlm.nih.gov/genbank/) databases. BioEdit software v7.2.5 was then used to visualize, edit and call single-nucleotide polymorphisms (SNPs) by comparison with the reference sequences.

Gene copy number amplifications were detected using previously published protocols [18, 26]. PCR amplification for *P. falciparum* plasmepsin-II (*pfpm2*), multi-drug resistance-I (*pfmdr1*), and β-tubulin (*pfβ-tubulin*) genes, was performed separately with the *pfβ-tubulin* gene serving as an endogenous control. All samples with estimated copy numbers > 1.5 were defined as containing multiple copies and repeated for confirmation.

Data were saved into Microsoft Excel to calculate prevalence of mutations and gene deletion analysis. Haplotypes were called after excluding samples where all the SNPs of interest could not be called and, in the case of multi-gene haplotypes, by excluding those samples from which only one of the genotyping assays were successful. The percentages of single nucleotide polymorphisms (SNPs) and haplotypes were calculated with a 95% confidence interval and were compared between the two rounds using the *z*-test.

### Ethics approvals

The protocol, patient information sheet and informed consent forms for this study were approved by the Oxford Tropical Medicine Ethics Committee at the University of Oxford (Reference 5108-16), Faculty of Tropical Medicine Ethics Committee at Mahidol University (Submission no. TMEC 16-060) and the Ministry of Health of the Republic of Chad (Reference 299/PR/PM/MESRS/SG/CNB/2016).

## Results

Sample collection rounds were conducted from September 2016 to January 2017 (Round 1) and from August to December 2017 (Round 2) with 187 and 211 subjects recruited in the respective rounds. The participants were predominantly > 18 year old males (394/398, 98.9%) (Table 1). Assay success rates in rounds 1 and 2 ranged from 84 to 91% and 82% to 95% respectively with the lowest success rates obtained from gene copy number assays.

**Table 1:** Study Subject demographics

Year	Both rounds	2016	2017
Total (N)	398	187	211
Male (%)	394 (98.9)	185 (98.9)	209 (99.1)
Female (%)	4 (1.1)	2 (1.1)	2 (0.9)
Age range * (overall, in years)	5—59	20—59	5—59

^*^ Age: 1 missing value in round 1, 2 in round 2

### Polymorphisms in *pfk13*

Mutations in the propeller domains of the *P. falciparum* gene (*pfk13*) encoding the Kelch13 protein first identified in South-East Asia are considered to be reliable markers of artemisinin resistance as defined by delayed parasite clearance following treatment [27]. In addition, a non-synonymous mutation (E252**Q**) upstream of codon 441, the first codon of the propeller domain, also appears to be associated with delayed parasite clearance but was only transiently observed in Myanmar and bordering areas in Thailand. For this study, the entire *pfk13* gene was sequenced and assessed for the presence of mutations. A polymorphism at codon 189 (K189**T**) was the most commonly observed over both sample collection rounds with 54.4% (95%CI 49.2–59.6%) of the parasite samples overall carrying this mutation (54.8% [95%CI 47.0–62.4%], 54.2 [95%CI 47.1–61.1%] in rounds 1, 2 respectively; see Table 2 and Table S2 for additional details). Only 4 of 349 successfully analysed samples (1.1%) had parasites with non-synonymous mutations in the propeller regions at codons A578S, Q633R, V636A, W660C. None of these mutations are known to be associated with artemisinin resistance.

**Table 2:** Mutations and haplotypes

Gene	Haplotype	Total		Year 2016		Year 2017	
		**Number**	**Percentage**	**Number**	**Percentage**	**Number**	**Percentage**
***PfKelch***	A578**S**	1	0.3	0	0	1	0.5
Total n = 349	K189**N**	7	2	6	3.8	1	0.5
	K189**N**/K	2	0.6	0	0	2	1
	K189**T**	159	45.6	76	48.4	83	43.2
	K189**T**, N197**D**/N	1	0.3	0	0	1	0.5
	K189**T**, V636**A**/V	1	0.3	0	0	1	0.5
	K189**T**, W660**C**	1	0.3	0	0	1	0.5
	K189**T**/K	25	7.2	8	5.1	17	8.9
	K189**T**/K, N197**D**/N	1	0.3	0	0	1	0.5
	K189**T**, I354**V**	1	0.3	1	0.6	0	0
	K189**T**, N197**D**	1	0.3	1	0.6	0	0
	L258**M**	1	0.3	1	0.6	0	0
	N195**D**/N	1	0.3	0	0	1	0.5
	N195**K**/N	1	0.3	0	0	1	0.5
	N197**D**	1	0.3	1	0.6	0	0
	Q633**R**	1	0.3	1	0.6	0	0
	R255**K**	11	3.2	6	3.8	5	2.6
	S213**G**/S	1	0.3	0	0	1	0.5
	WT	132	37.8	56	35.7	76	39.6
***Pfcrt***	CVMNK	296	84.8	129	83.8	167	85.6
Total n = 353	CV**IET**	19	5.4	11	7.1	8	4.1
Positions	CVMN/DK/**T**	3	0.9	0	0	7	3.6
72–76	CVMNK/**T**	10	2.9	10	6.5	0	0
	CVM/**I**NK/**T**	5	1.4	4	2.6	1	0.5
	CVM/**I**N/**D**K	2	0.6	0	0	2	1
	CVM/**I**N/**D**K/**T**	14	4	3	1.9	11	5.6
	CVM/**IET**, CVM/**IDT**, CV**ID**K, CVM/**I**N/**DT**	4	1.1	1	0.6	3	1.5
***Pfmdr1***	NYSND	134	39.9	54	36.7	80	42.3
Total n = 336	N**F**SND	160	47.6	71	48.3	89	47.1
Positions	NY/**F**SND	22	6.5	11	7.5	11	5.8
86, 184, 1034,	N/**YF**SND	1	0.3	0	0	1	0.5
1042, 1246	N/**Y**YSND	3	0.9	1	0.7	2	1.1
	N/**Y**Y/**F**SND	1	0.3	1	0.7	0	0
	**YF**SND	13	3.9	8	5.4	5	2.6
	**Y**YSND	2	0.6	1	0.7	1	0.5
***Pfmdr1_Pfcrt***	N**F**D + K	134	40.5	58	39.5	76	41.3
Total n = 331	N**F**D + K/**T**	15	4.5	8	5.4	7	3.8
*Pfmdr1*	N**F**D + **T**	9	2.7	5	3.4	4	2.2
positions	NYD + K	112	33.8	44	29.9	68	37
86, 184, 1246 *Pfcrt* position	NYD + K/**T**	11	3.3	6	4.1	5	2.7
76	NYD + **T**	9	2.7	4	2.7	5	2.7
	NY/**F**D + K	18	5.4	9	6.1	9	4.9
	NY/**F**D + K/T	4	1.2	2	1.4	2	1.1
	N/**Y**YD + K	2	0.6	1	0.7	1	0.5
	**YF**D + K	10	3	6	4.1	4	2.2
	**YF**D + T	2	0.6	2	1.4	0	0
	**YF**D + K/**T**, **Y**YD + K, **Y**YD + **T**, N/**YF**D + K, N/**Y**Y/**F**D + K	5	1.5	2	1.4	3	1.6
***Pfdhfr***	A**I**C**N**I	13	3.8	7	4.5	6	3.2
Total n = 346	A**I**C/**RN**I	6	1.7	0	0	6	3.2
Positions	A**IRN**I	275	79.5	125	80.1	150	78.9
16, 51, 59,	ANCSI	5	1.4	1	0.6	4	2.1
108, 164	AN**RN**I	31	9	12	7.7	19	10
	AN/**IRN**I	13	3.8	8	5.1	5	2.6
	ANC/**RN**I, AN/**I**CS/**N**I, AN/**I**C/**R**S/**N**I	3	0.9	3	1.9	0	0
***Pfdhps***	**A**AKAA	185	53.2	76	50.3	109	55.3
Total n = 348	AA/**G**KAA	11	3.2	2	1.3	9	4.6
Positions	**AG**KAA	24	6.9	13	8.6	11	5.6
436, 437, 540,	**AG**K**GS**	12	3.4	4	2.6	8	4.1
581, 613	SAKAA	5	1.4	3	2.0	2	1.0
	S**GEA**A	5	1.4	2	1.3	3	1.5
	S**GEG**A	10	2.9	3	2.0	7	3.6
	S**G**KAA	62	17.8	30	19.9	32	16.2
	S/**A**AKAA	9	2.6	8	5.3	1	0.5
	S/**A**A/**G**KAA	13	3.7	4	2.6	9	4.6
	S/**AG**KAA	4	1.1	3	2.0	1	0.5
	**A**A/**G**KA**S**, **AG**KA**S**, **AG**K/**E**AA, **C**AKAA, SAK/**EG**A, SA/**G**KAA, S**G**KA/**G**A, S/**A**A/**G**KA/**G**A	8	2.3	3	2	5	2.5
***Pfdhfr_Pfdhps***	A**I**C**N**I + **A**AKAA	8	2.4	3	2	5	2.7
Total n = 336	A**I**C**N**I + **AG**KAA	1	0.3	1	0.7	0	0
*Pfdhfr*	A**I**C**N**I + S**GE**AA	2	0.6	1	0.7	1	0.5
positions	A**I**C**N**I + S**G**KAA	1	0.3	1	0.7	0	0
16, 51, 59,	A**I**C**N**I + S/**A**AKAA	1	0.3	1	0.7	0	0
108, 164	A**I**C/**RN**I + **A**AKAA	3	0.9	0	0	3	1.6
*Pfdhps*	A**I**C/**RN**I + S/**A**A/**G**KAA	2	0.6	0	0	2	1.1
positions	A**I**C/**RN**I + S/**AG**KAA	1	0.3	0	0	1	0.5
436, 437, 540,	A**IRN**I + **A**AKAA	135	40.2	58	38.9	77	41.2
581, 613	A**IRN**I + **A**A/**G**KAA	10	3	2	1.3	8	4.3
	A**IRN**I + **A**A/**G**KA**S**	1	0.3	1	0.7	0	0
	A**IRN**I + **AG**KAA	20	6	10	6.7	10	5.3
	A**IRN**I + **AG**KA**S**	1	0.3	1	0.7	0	0
	A**IRN**I + **AG**K**GS**	10	3	4	2.7	6	3.2
	A**IRN**I + **AG**K/**E**AA	1	0.3	1	0.7	0	0
	A**IRN**I + **C**AKAA	1	0.3	0	0	1	0.5
	A**IRN**I + SAKAA	4	1.2	3	2	1	0.5
	A**IRN**I + SAK/**EG**A	1	0.3	0	0	1	0.5
	A**IRN**I + SA/**G**KAA	1	0.3	0	0	1	0.5
	A**IRN**I + S**GE**AA	2	0.6	0	0	2	1.1
	A**IRN**I + S**GEG**A	8	2.4	3	2	5	2.7
	A**IRN**I + S**G**KAA	53	15.8	27	18.1	26	13.9
	A**IRN**I + S**G**KA/**G**A	1	0.3	0	0	1	0.5
	A**IRN**I + S/**A**AKAA	7	2.1	6	4	1	0.5
	A**IRN**I + S/**A**A/**G**KAA	7	2.1	1	0.7	6	3.2
	A**IRN**I + S/**A**A/**G**KA/**G**A	1	0.3	0	0	1	0.5
	A**IRN**I + S/**AG**KAA	1	0.3	1	0.7	0	0
	ANCSI + **A**AKAA	3	0.9	1	0.7	2	1.1
	ANCSI + **AG**K**GS**	1	0.3	0	0	1	0.5
	ANCSI + S/**A**A/**G**KAA	1	0.3	0	0	1	0.5
	ANC/**RN**I + S/**AG**KAA	1	0.3	1	0.7	0	0
	AN**RN**I + **A**AKAA	20	6	7	4.7	13	7
	AN**RN**I + **AG**K**GS**	1	0.3	0	0	1	0.5
	AN**RN**I + SAKAA	1	0.3	0	0	1	0.5
	AN**RN**I + S**GE**AA	1	0.3	1	0.7	0	0
	AN**RN**I + S**GEG**A	1	0.3	0	0	1	0.5
	AN**RN**I + S**G**KAA	5	1.5	2	1.3	3	1.6
	AN**RN**I + S/**A**AKAA	1	0.3	1	0.7	0	0
	AN**RN**I + S/**AG**KAA	1	0.3	1	0.7	0	0
	AN/**I**CS/**N**I + **A**AKAA	1	0.3	1	0.7	0	0
	AN/**I**C/**R**S/**N**I + S/**A**A/**G**KAA	1	0.3	1	0.7	0	0
	AN/**IRN**I + **A**AKAA	10	3	6	4	4	2.1
	AN/**IRN**I + **AG**KAA	1	0.3	0	0	1	0.5
	AN/**IRN**I + S/**A**A/**G**KAA	2	0.6	2	1.3	0	0
***CytB* (**Y268**S)**	Y258	347	100	149	100	198	100
Total n = 347	Y268**S**	0	0	0	0	0	0
***Pfmdr1* CNV**	Single copy	335	99.7	151	100	184	99.5
Total n = 335	**Multiple copies**	1	0.3	0	0	1	0.5
***Pfpm2* CNV**	Single copy	339	100	152	100	187	100
Total n = 340	**Multiple copies**	0	0	0	0	0	0

Mutants are shown as Axxx**B** where ‘A’ refers to the amino acid in single-letter code encoded by the wild type codon, the number ‘xxx’ indicates the codon position and ‘**B**’ is the amino acid encoded by the mutant allele. Mixed infections are indicated as A/**B**. Similarly for haplotypes, amino acids encoded by the wild type codons are shown in black and mutants are in underlined bold

### SP resistance markers

Nearly all parasites had at least one mutation in the *P. falciparum* dihydrofolate reductase (*pfdhfr*) gene with only 5/346 (1.4%, 95%CI 0.5–3.3%) parasites carrying wild type alleles. A large majority (285/336, 84.8% [95%CI 80.5–88.5%] overall) of the parasites were ‘triple’ mutants with the 51**I**-59**R**-108** N** haplotype (See Table 2 for a detailed list of the mutations and haplotypes). Similarly, nearly all of parasite samples indicated the presence of one or more mutations in the *P. falciparum* dihydropteroate synthase gene (*pfdhps*) with only 5 of 348 (1.5% [95%CI 0.5–3.4%]) samples carried wild type alleles. The most common haplotype was a single mutation at position 436 (S436**A/C**) and overall 53.4% (186/348, 95%CI 48.0–58.8%) of the parasites carried this mutation. ‘Triple’ mutations of *pfdhps* 437**G**-540**E**-581**G**, known to be associated with reduced effectiveness of IPTp when part of a ‘sextuple’ mutation haplotype of *pfdhfr*-*pfdhps* genes, were rare (8/336 overall, 2.4% [95%CI 1.2–4.6%]; see Additional file 2: Table 2) [28].

### Markers of resistance to lumefantrine, amodiaquine, chloroquine

Polymorphisms in *pfmdr1 (*codons 86, 184, 1246), particularly when associated with another in the chloroquine resistance transporter gene (*pfcrt*; codon 76) have been shown to be associated with recrudescence of parasites following treatment with AL and ASAQ [20]. The *pfmdr1* haplotype N86-184**F**-D1246 + *pfcrt* K76 (N**F**D-K) is selected in recrudescent infections detected after treatment with AL whereas the inverse haplotype 86**Y**-Y184-1246**Y** + 76** T** (YYY-T) is selected by ASAQ. Approximately half of all isolates (172/331, 52.0% [95%CI 46.4–57.5%] overall) were found to have the NFD-K haplotype whereas none had the **Y**Y**Y**-**T** haplotype and only a small minority (20/336, 6.0% [95%CI 3.7–9.0%]) carried the 86**Y** mutation. As is increasingly being observed at multiple locations across the African continent [29], parasites with the *pfcrt* K76 wild type allele were predominant over both rounds of the study (299/353, 84.7% [95%CI 80.5–88.3%]) (Table 2).

### Other molecular markers of anti-malarial drug resistance

In addition to the molecular markers described above, the samples were also assessed for mutations in the *P. falciparum* cytochrome B (*pfcytB*) gene and for copy number amplifications of *pfmdr1* and *pfpm2*. Increased copy numbers of *pfmdr1* and *pfpm2* have been shown to be strongly associated with treatment failures with mefloquine and piperaquine respectively. Further, atovaquone-proguanil, more commonly known under its trade name Malarone® is also an important prophylactic drug prescribed to travellers, resistance to which is conferred by a single mutation in the *pfcytB* gene (268**S**). These drugs are not commonly used in African countries for treatment but mefloquine and Malarone are often used for prophylaxis and piperaquine in combination with dihydroartemisinin is being considered as a replacement for SP in IPTp as well as a first-line treatment. Overall, genotyping assays for *pfmdr1*, *pfpm2* copy numbers and *pfcytB* were successful from 335, 339 and 347 samples respectively in which only one isolate carrying an amplification in the *pfmdr1* gene was detected (Additional file 2).

### Detection of *pfhrp2/3* gene deletions

In round 2 of sample collection, all symptomatic individuals who were negative with the PfHRP2-based RDT were re-tested with an RDT which detected *P. falciparum* lactate dehydrogenase (PfLDH) protein. Of the 211 subjects recruited in round 2, only 1 patient had discordant RDT results, i.e., a negative result from the PfHRP2 RDT but positive with the PfLDH RDT. Neither the sample collected from this patient nor any of those collected in round 2 showed any deletions in the *pfhrp2/3* genes which have previously been shown to result in reduced or no production of the encoded protein (see Additional file 2).

## Discussion

The first pillar of the strategic framework described in the WHO Global Technical Strategy for Malaria is ensuring universal access to malaria prevention, prompt diagnosis and effective treatment. The effectiveness of chemoprevention and treatments in particular is heavily dependent on the efficacy of the drugs and the accuracy of the diagnostic tools used to target the treatments. In the absence of data from TES, molecular surveillance can supplement the geographic coverage of drug efficacy monitoring and help with targeting TES to locations where an increased prevalence of resistance markers is detected. This is particularly relevant in regions or countries from where few data are available. This study was performed in southern Chad where neither studies to assess the efficacy of the artemisinin-based combination used as first-line treatments nor on the prevalence of drug resistance markers have been published [25]. It is important to note that this study was conducted at a private clinic serving a specific sub-population of patients and hence the allele frequencies reported here may not directly reflect those in the general population from the region where the study was conducted.

The emergence of artemisinin resistance in South-East Asia and more recently in Rwanda is a major threat to malaria control and elimination efforts. It can only be confirmed when in vitro or in vivo phenotypes indicative of resistance have been detected. Some mutations in the propeller region of the *pfk13* gene appear to be reliable predictors of the resistance phenotype given that this association has been observed in independently emergent artemisinin resistance in Africa and South America [9, 30], i.e., outside of the region where they were first observed. In the current study, none of the mutations validated or identified by the WHO or from the WWARN pooled analysis as being associated with artemisinin resistance were observed. The K189**T** polymorphism, which is not associated with delayed parasite clearance [27], was the most frequently detected allele as has also been reported in other studies conducted across Africa and elsewhere [30–33]. This allele may not be under artemisinin selective pressure (see Table 3) and it appears more likely that it is an ‘alternative wild type’ in some parasite populations. This hypothesis could be verified if this polymorphism is detected in samples collected before ACTs were widely deployed across Africa.

**Table 3:** Yearly prevalence of selected markers

YEAR	Both rounds			2016			2017			*p*-value (*z* test)
	**n/N**	**%**	**95% CI**	**n/N**	**%**	**95% CI**	**n/N**	**%**	**95% CI**	
*pfk13* K189T	190/349	54.4%	49.2–59.6%	86/157	54.8%	47.0–62.4%	104/192	54.2%	47.1–61.1%	0.909
*pfmdr1* + *pfcrt* NFD + ﻿K	172/331	52.0%	46.4–57.5%	77/147	53.1%	44.3–60.3%	95/184	51.1%	44.5–58.7%	0.892
*pfdhfr* IRN	285/336	84.8%	80.5–88.5%	127/149	85.2%	78.7–90.0%	158/187	84.5%	78.6–89.0%	0.850

Artemether-lumefantrine is the single most widely used artemisinin-based combination across the malaria endemic world, especially in Africa [25, 34]. It has mostly proven to be efficacious over the nearly 15 years since ACT was recommended as first-line treatments worldwide and across the malaria endemic world. Recent reports of reduced efficacy from Angola, Burkina Faso and the Democratic Republic of Congo however underline the need for continued surveillance of the efficacy of this drug [35–37]. In southern Chad, a relatively high prevalence of the combined *pfmdr1*-*pfcrt* N**F**D-K haplotype was detected which remained unchanged over both rounds of sample collection (see Table 3). The AL combination is likely to retain its efficacy given the likely very high rate of parasite killing by the artemisinin component aided by partial immunity in the human hosts. This result could nonetheless be considered as a signal to trigger a TES to verify the efficacy of AL in the region. If such a TES does indeed confirm a reduction in AL efficacy, a ready solution would be available. The efficacy of ASAQ, also a first-line treatment per the national policy, is likely to be very high in this context given the low prevalence of the 86Y allele and the complete absence of the *pfmdr1*-*pfcrt* **Y**Y**Y**-**T** combined haplotype in the sampled population.

The prevalence of the alleles or haplotypes associated with reduced susceptibility to amodiaquine could have been expected to be high given the implementation of SMC in Chad and the consequent amodiaquine drug pressure. Given the low prevalence of haplotypes associated with amodiaquine treatment failures, SMC can also be expected to be effective despite the high prevalence of parasites with mutations in the *pfdhfr* and *pfdhps* genes if implemented with high coverage and adherence to the recommended 3-day regimen. Continued implementation of SMC must however be accompanied by continued monitoring of the effectiveness of the intervention and of the prevalence of the markers of SP resistance. Similarly IPTp with SP is also likely to retain its effectiveness given that it only appears to drop in zones where parasites with the quintuple or sextuple combined haplotypes are highly prevalent [38]. Here too, continued surveillance of the effectiveness of the intervention alongside molecular marker prevalence surveys should be the norm to be able to detect increasing prevalence of such haplotypes and hence to determine whether alternative drugs and/or strategies are needed [39].

Current standard protocols for screening and confirming the presence of isolates with *pfhrp2/3* deletions require the use of microscopy to confirm *P. falciparum* infections [40]. Reliable microscopy however is not consistently available, as was the case at this study site, so a novel approach to screening for such deletions was piloted in the second round of sample collection. An RDT not dependent on PfHRP2 for the detection of *P. falciparum* infections was used in cases where the first RDT was negative in an attempt to identify cases which may have these deletions. Only one such case with discordant RDT results was detected which eventually did not appear to have these deletions in any case. These results must however be interpreted with caution. Firstly, in the absence of expert microscopy it is impossible to eliminate the possibility that there were infections with parasites carrying *pfhrp2/3* deletions at a low enough parasite density that they could not be detected by the PfLDH RDT. Second, in the one case where the RDT results were discordant or even in some of the others, it is possible that there were multiple infecting parasite strains, only some of which had the deletions. Mutated parasites in such mixed infections are masked and cannot be detected by currently recommended protocols which rely on the *absence* of PCR products to detect deletions. This does not undermine the rationale of using PfLDH RDTs as a screening tool for false negative PfHRP2-RDT results caused by *pfhrp2/3* gene deletions. This approach does however need further validation and perhaps to be applied with newer PCR protocols which may be able to detect parasites with deletions even in mixed infections.

## Conclusion

The study reported provides valuable data on the likely efficacy of preventive and curative treatments used in Chad. Given that this study was conducted in a very specific sub-population, the prevalences reported here may not exactly reflect those in the general population but nonetheless adds to the very sparse information currently available from Chad. The prevalence of the mutations studied here remained mostly unchanged over the two rounds of sample collection. This indicates a stable parasite population in which *pfdhfr* triple mutations are near fixation and with a relatively high prevalence of mutations associated with reduced lumefantrine susceptibility. The reported data are from specimens collected more than 3 years ago so there may have been changes in the prevalence of the polymorphisms in the intervening period. These findings further emphasize the need for continued monitoring and surveillance of the efficacy and effectiveness of the malaria control interventions, specifically a TES to verify the efficacy of AL.

## Supplementary Information

**Additional file 1: Data S1**. Primer sequences and protocols used in this study.

**Additional file 2: Data S2.** Individual sample results.

## Data Availability

All data generated or analysed during this study are included in this published article and its supplementary information files.

## Abbreviations

ACT: Artemisinin Combination Therapy
ART-R: Artemisinin resistance
CNV: Copy number variation
CRF: Case Report Form
DNA: Deoxyribonucleic acid
*pfcrt*: *P. falciparum* Chloroquine resistance transporter gene
*pfCYTb*: *P. falciparum* Cytochrome B gene
*pfdhfr*: *P. falciparum* Dihydrofolate reductase gene
*pfdhps*: *P. falciparum* Dihydropteroate synthase gene
*pfk13*: *P. falciparum* Kelch 13 gene
*pfmdr1*: *P. falciparum* Multi-drug resistance-1 gene
*pfpm2/3*: *P. falciparum* Plasmepsin 2/3 gene
SNP: Single nucleotide polymorphism
SOP: Standard Operating Procedure
WHO: World Health Organization
WWARN: WorldWide Antimalarial Resistance Network
