# Supplementary material for: Molecular surveillance for operationally relevant genetic polymorphisms in Plasmodium falciparum in Southern Chad, 2016–2017
Source: Malar J. 2022 Mar 12;21:83. doi: 10.1186/s12936-022-04095-9 (PMC8917628; doi:10.1186/s12936-022-04095-9)
Supplement: Supplementary file 1 — Additional file 1: Data S1. Primer sequences and protocols used in this study. [file 12936_2022_4095_MOESM1_ESM.docx]

Supplementary data S1: Primer sequences and protocols used in this study

| Gene | | Sequence (5' -> 3') | Ref |
| --- | --- | --- | --- |
| Pfkelch13 | Single | TGGAAGGAGAAAAAGTAAAAACAAAA | Ashley EA et al., 2014 |
|  |  | TGTGCATGAAAATAAATATTAAAGAAG |  |
| PfCRT SNPs at codons 72-76 | Nest 1 | CCGTTAATAATAAATACACGCAG | Djimde A. et al., 2001 |
|  |  | CGGATGTTACAAAACTATAGTTACC |  |
|  | Nest 2 | TGTGCTCATGTGTTTAAACTT |  |
|  |  | CAAAACTATAGTTACCAATTTTG |  |
| Pfmdr1 SNPs at codon 86 and 184 | Nest 1 | TGTTGAAAGATGGGTAAAGAGCAGAAAGAG | Duraisingh, M. T. et al., 1997 |
|  |  | TACTTTCTTATTACATATGACACCACAAACA |  |
|  | Nest 2 | AAAGATGGTAACCTCAGTATCAAAGAAGAG |  |
|  |  | TACTTTCTTATTACATATGACACCACAAACA |  |
| Pfdhfr | Nest 1 | ATGGARSAMSTYTSMGABGTWTTY | Duraisingh, M. T. et al., 1998 |
|  |  | AGTATATACATCGCTAACAGA |  |
|  | Nest 2 | TSMGABGTWTTYGAYATTTAYGC |  |
|  |  | AGTATATACATCGCTAACAGA |  |
| Pfdhps | Nest 1 | GTTGAACCTAAACGTGCTGT | Duraisingh, M. T. et al., 1998 |
|  |  | AATTGTGTGATTTGTCCACAA |  |
|  | Nest 2 | AACCTAAACGTGCTGTTCAA |  |
|  |  | AATTGTGTGATTTGTCCACAA |  |
| PfcytB | Nest 1 | AATTATGATATTTATTGTAACTGC | Ekala MT. et al., 2007 |
|  |  | ACAGAATAATCTCTAGCACC |  |
| Pfmdr1 Copy Number Variations |  | TGCATCTATAAAACGATCAGACAAA | Price RN. et al., 2004 |
|  |  | TCGTGTGTTCCATGTGACTGT |  |
|  | Real time | FAM-TTTAATAACCCTGATCGAAATGGAACCTTTG |  |
|  |  | AAAAATATGATGTGCGCAAGTGA |  |
|  |  | AACTTCCTTTGTGGACATTCTTCCT |  |
|  |  | VIC-TAGCACATGCCGTTAAATATCTTCCATGTCT |  |
| Pfplasmepsin2 Copy Number Variations |  | CCACTTGTGGTAACACGAAATTA | Ansbro M. et al., 2020 |
|  |  | TGGTTCAAGGTATTGTTTAGGTTC |  |
|  | Real time | FAM-CAGGATCTGCTAATTTATGGGTCCCA |  |
|  |  | TGATGTGCGCAAGTGATCC |  |
|  |  | TCCTTTGTGGACATTCTTCCTC |  |
|  |  | VIC-CACATGCCGTTAAATATCTTCCATGTCT |  |
| Pfhrp2 exon 2 | Nest 1 | CAAAAGGACTTAATTTAAATAAGAG | Baker J. et al., 2005 |
| PF3D7_0831800 |  | AATAAATTTAATGGCGTAGGCA |  |
| MAL7P1.231 | Nest 2 | ATTATTACACGAAACTCAAGCAC |  |
|  |  | AATAAATTTAATGGCGTAGGCA |  |
| Pfhrp2 upstream | Nest 1 | GATATCATTAGAAAACAAGAGCTTAG | Baker J. et al., 2005 |
| PF3D7_0831900 |  | TATCCAATCCTTCCTTTGCAACACC |  |
| MAL7P1.230 | Nest 2 | TATGAACGCAATTTAAGTGAGGCAG |  |
|  |  | TATCCAATCCTTCCTTTGCAACACC |  |
| Pfhrp2 downstream | Nest 1 | AGACAAGCTACCAAAGATGCAGGTG | Baker J. et al., 2005 |
| PF3D7_0831700 |  | TAAATGTGTATCTCCTGAGGTAGC |  |
| MAL7P1.228 | Nest 2 | CCATTGCTGGTTTAAATGTTTTAAG |  |
|  |  | TAAATGTGTATCTCCTGAGGTAGC |  |
| Pfhrp3 exon 2 | Nest 1 | AATGCAAAAGGACTTAATTC | Baker J. et al., 2005 |
| PF3D7_1372200 |  | TGGTGTAAGTGATGCGTAGT |  |
| MAL13P1.480 | Nest 2 | AAATAAGAGATTATTACACGAAAG |  |
|  |  | TGGTGTAAGTGATGCGTAGT |  |

Duraisingh MT, Curtis J, Warhurst DC. [Plasmodium falciparum: detection of polymorphisms in the dihydrofolate reductase and dihydropteroate synthetase genes by PCR and restriction digestion.](https://pubmed.ncbi.nlm.nih.gov/9603482/) Exp Parasitol. 1998 May;89(1):1-8. doi: 10.1006/expr.1998.4274. PMID: 9603482

[M T Duraisingh](https://pubmed.ncbi.nlm.nih.gov/?sort=date&term=Duraisingh+MT&cauthor_id=9075340), [C J Drakeley](https://pubmed.ncbi.nlm.nih.gov/?sort=date&term=Drakeley+CJ&cauthor_id=9075340), [O Muller](https://pubmed.ncbi.nlm.nih.gov/?sort=date&term=Muller+O&cauthor_id=9075340), [R Bailey](https://pubmed.ncbi.nlm.nih.gov/?sort=date&term=Bailey+R&cauthor_id=9075340), [G Snounou](https://pubmed.ncbi.nlm.nih.gov/?sort=date&term=Snounou+G&cauthor_id=9075340), [G A Targett](https://pubmed.ncbi.nlm.nih.gov/?sort=date&term=Targett+GA&cauthor_id=9075340), [B M Greenwood](https://pubmed.ncbi.nlm.nih.gov/?sort=date&term=Greenwood+BM&cauthor_id=9075340), [D C Warhurst](https://pubmed.ncbi.nlm.nih.gov/?sort=date&term=Warhurst+DC&cauthor_id=9075340). Evidence for selection for the tyrosine-86 allele of the pfmdr 1 gene of Plasmodium falciparum by chloroquine and amodiaquine Parasitology 1997 Mar;114 ( Pt 3):205-11. doi: 10.1017/s0031182096008487

Ekala MT, Khim N, Legrand E, Randrianarivelojosia M, Jambou R, Fandeur T, Menard D, Assi SB, Henry MC, Rogier C, Bouchier C, Mercereau-Puijalon O. [Sequence analysis of Plasmodium falciparum cytochrome b in multiple geographic sites.](https://pubmed.ncbi.nlm.nih.gov/18086297/) Malar J. 2007 Dec 17;6:164. doi: 10.1186/1475-2875-6-164.

[Ansbro](https://pubmed.ncbi.nlm.nih.gov/?sort=date&term=Ansbro+MR&cauthor_id=32404110)  MR, [Christopher G Jacob](https://pubmed.ncbi.nlm.nih.gov/?sort=date&term=Jacob+CG&cauthor_id=32404110) , [Roberto Amato](https://pubmed.ncbi.nlm.nih.gov/?sort=date&term=Amato+R&cauthor_id=32404110), [Mihir Kekre](https://pubmed.ncbi.nlm.nih.gov/?sort=date&term=Kekre+M&cauthor_id=32404110), [Chanaki Amaratunga](https://pubmed.ncbi.nlm.nih.gov/?sort=date&term=Amaratunga+C&cauthor_id=32404110) , [Sokunthea Sreng](https://pubmed.ncbi.nlm.nih.gov/?sort=date&term=Sreng+S&cauthor_id=32404110) , [Seila Suon](https://pubmed.ncbi.nlm.nih.gov/?sort=date&term=Suon+S&cauthor_id=32404110) , [Olivo Miotto](https://pubmed.ncbi.nlm.nih.gov/?sort=date&term=Miotto+O&cauthor_id=32404110) , [Rick M Fairhurst](https://pubmed.ncbi.nlm.nih.gov/?sort=date&term=Fairhurst+RM&cauthor_id=32404110) , [Thomas E Wellems](https://pubmed.ncbi.nlm.nih.gov/?sort=date&term=Wellems+TE&cauthor_id=32404110) , [Dominic P Kwiatkowski](https://pubmed.ncbi.nlm.nih.gov/?sort=date&term=Kwiatkowski+DP&cauthor_id=32404110). Development of copy number assays for detection and surveillance of piperaquine resistance associated plasmepsin 2/3 copy number variation in *Plasmodium falciparum* Malaria Journal 2020 May 13;19(1):181. doi: 10.1186/s12936-020-03249-x.

Baker J, Gatton ML, Peters J, Ho MF, McCarthy JS, Cheng Q. Transcription and expression of Plasmodium falciparum histidine-rich proteins in different stages and strains: implications for rapid diagnostic tests. PloS one. 2011;6(7):e22593.

Price RN, Uhlemann AC, Brockman A, McGready R, Ashley E, Phaipun L, Patel R, Laing K, Looareesuwan S, White NJ, Nosten F, Krishna S. [Mefloquine resistance in Plasmodium falciparum and increased pfmdr1 gene copy number.](https://pubmed.ncbi.nlm.nih.gov/15288742/) Lancet. 2004 Jul 31-Aug 6;364(9432):438-447. doi: 10.1016/S0140-6736(04)16767-6. PMID: 15288742
