# Supplementary material for: Molecular surveillance for operationally relevant genetic polymorphisms in Plasmodium falciparum in Southern Chad, 2016–2017
Source: Malar J. 2022 Mar 12;21:83. doi: 10.1186/s12936-022-04095-9 (PMC8917628; doi:10.1186/s12936-022-04095-9)
Supplement: Supplementary file 2 — Additional file 2: Data S2. Individual sample results. [file 12936_2022_4095_MOESM2_ESM.docx]

Supplementary data S2: Individual sample results

|  |  | *Pfkelch* | *Pfdhfr* | | | | | *Pfdhps* | | | | | *Pfmdr1* | | | | | *Pfcrt* | | | | | *Pfmdr1* CNV | *Pfplasmepsin2* CNV | *Pfcytochrome B* |
| --- | --- | --- | --- | --- | --- | --- | --- | --- | --- | --- | --- | --- | --- | --- | --- | --- | --- | --- | --- | --- | --- | --- | --- | --- | --- |
| No | Year |  | A16V | N51I | C59R | S108N | I164L | S436A | A437G | K540E/N | A581G | A613S | N86Y | Y184F | S1034C | N1042D | D1246Y | C72S | V73 | M74I | N75E/D | K76T |  |  | Y268S |
| 1 | 2016 | WT | A | I | R | N | I | A | G | K | A | A | N | Y | S | N | D | C | V | M | N | K | 1.16 | 0.86 | Y |
| 2 | 2016 | K189N | A | I | R | N | I | A | A | K | A | A | N | F | S | N | D | C | V | M | N | K/T | 0.97 | 0.94 | Y |
| 3 | 2016 | K189N | A | I | R | N | I | A | A | K | A | A | N | F | S | N | D | C | V | M | N | K | 1.00 | 0.86 | Y |
| 4 | 2016 | WT | A | I | R | N | I | A | A | K | A | A | N | Y | S | N | D | C | V | M | N | K | 0.87 | 0.92 | Y |
| 5 | 2016 | WT | A | N/I | R | N | I | A | A | K | A | A | N | Y/F | S | N | D | C | V | M | N | K | 1.03 | 1.00 | Y |
| 6 | 2016 | WT | A | I | R | N | I | A | A | K | A | A | N | Y | S | N | D | C | V | M | N | K | 0.72 | 0.85 | Y |
| 7 | 2016 | WT | A | I | R | N | I | A | A | K | A | A | N | Y/F | S | N | D | C | V | M | N | K | 1.11 | 1.06 | ND |
| 8 | 2016 | K189T | A | I | R | N | I | S | G | K | A | A | N | F | S | N | D | C | V | M | N | K | 0.91 | 0.84 | Y |
| 9 | 2016 | K189T | A | I | C | N | I | S/A | A | K | A | A | N | F | S | N | D | C | V | M | N | K | 1.09 | 1.00 | Y |
| 10 | 2016 | K189T | A | I | R | N | I | A | A | K | A | A | N | F | S | N | D | C | V | M | N | K | 0.88 | 0.89 | Y |
| 11 | 2016 | K189T/K | A | I | R | N | I | S/A | A | K | A | A | N | Y | S | N | D | C | V | M | N | K | 0.92 | 1.02 | Y |
| 12 | 2016 | R255K | A | I | R | N | I | S | G | K | A | A | N | F | S | N | D | C | V | M | N | K | 0.97 | 1.00 | Y |
| 13 | 2016 | K189T | A | I | R | N | I | A | G | K | A | A | N | Y | S | N | D | C | V | M/I | N/(A/G,A,A/T)* | K/T | 0.91 | 0.95 | Y |
| 15 | 2016 | R255K | A | I | R | N | I | A | A | K | A | A | N | F | S | N | D | C | V | M | N | K | 1.01 | 0.95 | Y |
| 16 | 2016 | WT | A | N | R | N | I | S/A | A | K | A | A | N | ND | S | N | D | C | V | M | N | K | 1.28 | 0.53 | ND |
| 17 | 2016 | WT | A | I | R | N | I | A | A | K | A | A | N | F | S | N | D | C | V | M | N | K | 1.00 | 0.94 | Y |
| 18 | 2016 | R255K | A | I | R | N | I | A | A | K | A | A | N | Y | S | N | D | C | V | M | N | K | 1.02 | 0.97 | Y |
| 19 | 2016 | WT | A | I | R | N | I | S | G | E | G | A | N | F | S | N | D | C | V | M | N | K | 1.02 | 0.93 | Y |
| 20 | 2016 | K189T | A | I | R | N | I | S | G | K | A | A | N | F | S | N | D | C | V | M | N | K | 1.28 | 0.93 | Y |
| 21 | 2016 | K189N | A | I | R | N | I | A | A | K | A | A | N | Y | S | N | D | C | V | M | N | K | 1.23 | 0.96 | Y |
| 22 | 2016 | K189T | A | I | R | N | I | A | A | K | A | A | N | F | S | N | D | C | V | M | N | K | 1.14 | 0.89 | Y |
| 23 | 2016 | K189T | A | I | R | N | I | S/A | A | K | A | A | N | F | S | N | D | C | V | M | N | K | 1.14 | 0.92 | Y |
| 24 | 2016 | K189T | A | N | R | N | I | A | A | K | A | A | N | F | S | N | D | C | V | M | N | K | 1.12 | 0.94 | Y |
| 25 | 2016 | K189T | A | I | R | N | I | A | A | K | A | A | N | Y | S | N | D | C | V | M | N | K | 1.12 | 0.98 | Y |
| 28 | 2016 | K189T | A | I | R | N | I | S | G | K | A | A | N | Y | S | N | D | C | V | I | E | T | 0.75 | 0.93 | Y |
| 29 | 2016 | WT | A | N | C/R | N | I | S/A | G | K | A | A | N | Y | S | N | D | C | V | M | N | K | 0.83 | 0.84 | Y |
| 30 | 2016 | WT | A | I | C | N | I | S | G | K | A | A | N | F | S | N | D | C | V | I | E | T | 0.85 | 0.86 | Y |
| 31 | 2016 | WT | A | I | R | N | I | A | A | K | A | A | N | F | S | N | D | C | V | M/I | N/D | K/T | 0.78 | 0.87 | Y |
| 32 | 2016 | K189T | A | I | R | N | I | A | A | K | A | A | N | F | S | N | D | C | V | M | N | K | 0.75 | 0.88 | Y |
| 33 | 2016 | K189T | A | I | R | N | I | A | A | K | A | A | N | Y | S | N | D | C | V | I | E | T | 0.89 | 0.87 | Y |
| 34 | 2016 | R255K | A | I | C | N | I | A | A | K | A | A | N | Y | S | N | D | C | V | M | N | K | 0.66 | 0.91 | Y |
| 35 | 2016 | N197D | A | I | R | N | I | A | A | K | A | A | N | Y | S | N | D | C | V | M | N | K | 0.74 | 1.23 | Y |
| 36 | 2016 | WT | A | I | R | N | I | S | A | K | A | A | N | F | S | N | D | C | V | M | N | K | 0.73 | 0.87 | Y |
| 37 | 2016 | K189T | A | N | R | N | I | A | A | K | A | A | N | Y | S | N | D | C | V | M | N | K | 1.03 | 0.96 | Y |
| 38 | 2016 | WT | A | I | R | N | I | S/A | A | K | A | A | N | Y | S | N | D | C | V | M | N | K | 0.91 | 0.76 | Y |
| 39 | 2016 | K189T | A | I | R | N | I | S | G | E | G | A | N | Y | S | N | D | C | V | M | N | K | 1.22 | 0.91 | Y |
| 40 | 2016 | K189T/K | A | I | R | N | I | A | A | K | A | A | N | F | S | N | D | C | V | M | N | K | 1.17 | 0.91 | Y |
| 41 | 2016 | K189T | ND | ND | ND | ND | ND | A | G | K | A | A | N | F | S | N | D | C | V | M | N | K | ND | ND | Y |
| 42 | 2016 | WT | A | I | R | N | I | A | G | K | G | S | N | Y | S | N | D | C | V | M | N | K | 1.36 | 0.87 | Y |
| 43 | 2016 | K189T | A | N | C | S | I | A | A | K | A | A | N | F | S | N | D | C | V | M | N | K | 1.15 | 0.99 | Y |
| 45 | 2016 | WT | A | I | R | N | I | A | A | K | A | A | N | F | S | N | D | C | V | M/I | N/D | K/T | 1.88 | 1.04 | Y |
| 46 | 2016 | K189T | ND | ND | ND | ND | ND | A | G | K | A | A | N | Y | S | N | D | C | V | M | N | K | ND | ND | Y |
| 48 | 2016 | K189T | A | I | R | N | I | S | G | K | A | A | N | F | S | N | D | C | V | M | N | K | 1.09 | 0.85 | Y |
| 49 | 2016 | K189T | A | I | R | N | I | A | A | K | A | A | N | F | S | N | D | C | V | M | N | K | 1.21 | 0.87 | Y |
| 51 | 2016 | WT | A | I | R | N | I | S/A | A/G | K | A | A | N | F | S | N | D | C | V | M | N | K | 1.25 | 0.77 | Y |
| 52 | 2016 | K189T | A | I | R | N | I | A | A | K | A | A | N | F | S | N | D | C | V | M | N | K | 0.99 | 0.82 | Y |
| 53 | 2016 | WT | A | I | R | N | I | S | G | K | A | A | Y | F | S | N | D | C | V | I | E | T | 1.27 | 0.70 | Y |
| 54 | 2016 | WT | A | I | R | N | I | A | A | K | A | A | N | F | S | N | D | C | V | M | N | K | 1.15 | 0.72 | Y |
| 55 | 2016 | WT | A | I | C | N | I | S | G | E | A | A | N | Y | S | N | D | C | V | M | N | K | 0.44 | 0.76 | Y |
| 56 | 2016 | WT | A | I | R | N | I | S | G | K | A | A | N | F | S | N | D | C | V | M | N | K | 1.23 | 0.72 | Y |
| 57 | 2016 | WT | A | I | R | N | I | A | G | K | A | A | N | Y/F | S | N | D | C | V | M/I | N/(A/G,A,A/T)* | K/T | 1.03 | 0.84 | Y |
| 58 | 2016 | K189T | A | I | R | N | I | A | G | K | A | A | N | F | S | N | D | C | V | M | N | K | 1.11 | 0.75 | Y |
| 59 | 2016 | WT | A | I | R | N | I | S | G | K | A | A | N | F | S | N | D | C | V | M | N | K/T | 0.94 | 0.74 | Y |
| 60 | 2016 | WT | A | N/I | C | S/N | I | A | A | K | A | A | N | Y/F | S | N | D | C | V | M | N | K | 1.08 | 0.87 | Y |
| 61 | 2016 | K189T | A | I | R | N | I | S | G | K | A | A | N | Y | S | N | D | C | V | M | N | K | 1.12 | 0.83 | Y |
| 62 | 2016 | WT | A | I | R | N | I | A | G | K | G | S | N | F | S | N | D | C | V | M | N | K/T | 0.99 | 0.90 | Y |
| 63 | 2016 | K189T | A | I | R | N | I | S | G | E | G | A | N | F | S | N | D | C | V | M | N | K/T | 1.03 | 0.82 | ND |
| 64 | 2016 | WT | A | I | R | N | I | A | A | K | A | A | N | Y | S | N | D | C | V | M | N | K | 1.06 | 0.77 | Y |
| 65 | 2016 | K189T | A | I | R | N | I | A | G | K | A | S | Y | Y | S | N | D | C | V | M | N | K | 1.16 | 0.91 | Y |
| 66 | 2016 | K189N | A | I | R | N | I | A | A | K | A | A | N | F | S | N | D | C | V | M | N | K | 1.14 | 0.86 | Y |
| 67 | 2016 | WT | A | I | R | N | I | ND | ND | ND | ND | ND | ND | ND | ND | ND | ND | ND | ND | ND | ND | ND | ND | ND | ND |
| 68 | 2016 | K189T | A | I | R | N | I | S | G | K | A | A | N | Y | S | N | D | C | V | M | N | K | 0.79 | 0.83 | Y |
| 69 | 2016 | ND | A | I | R | N | I | ND | ND | ND | ND | ND | ND | ND | ND | ND | ND | ND | ND | ND | ND | ND | ND | ND | Y |
| 70 | 2016 | K189T | A | I | R | N | I | A | A | K | A | A | N | Y | S | N | D | C | V | M | N | K | 0.73 | 0.81 | Y |
| 71 | 2016 | K189T | A | I | R | N | I | S | G | K | A | A | N | Y | S | N | D | C | V | M | N | K | 0.95 | 1.18 | Y |
| 72 | 2016 | K189T | A | I | R | N | I | S/A | A | K | A | A | N | F | S | N | D | C | V | M | N | K | ND | 0.95 | Y |
| 73 | 2016 | K189T | A | I | R | N | I | S | G | K | A | A | N | Y | S | N | D | C | V | M | N | K | 1.12 | 0.94 | Y |
| 74 | 2016 | K189T | A | N | R | N | I | A | A | K | A | A | N | F | S | N | D | C | V | M/I | N/(A/G,A,A/T)* | K/T | 1.23 | 0.89 | Y |
| 75 | 2016 | WT | A | I | R | N | I | S | G | K | A | A | N | F | S | N | D | C | V | M | N | K | 1.09 | 0.92 | Y |
| 76 | 2016 | K189T | A | I | R | N | I | A | G | K | A | A | Y | F | S | N | D | C | V | M | N | K | 1.14 | 0.95 | Y |
| 77 | 2016 | K189T | A | I | R | N | I | A | A | K | A | A | N | Y | S | N | D | C | V | M | N | K | 1.22 | 0.88 | Y |
| 78 | 2016 | K189T+I354V | A | N | R | N | I | S | G | K | A | A | N | F | S | N | D | C | V | M | N | K | 1.01 | 0.84 | Y |
| 80 | 2016 | K189T | A | N | R | N | I | A | A | K | A | A | N | Y | S | N | D | C | V | M | N | K | 1.05 | 1.00 | Y |
| 81 | 2016 | K189T | A | I | R | N | I | S | G | K | A | A | N | F | S | N | D | C | V | M | N | K | 0.62 | 0.75 | Y |
| 82 | 2016 | K189T/K | A | I | R | N | I | A | ND | K | A | A | N | F | S | N | D | C | V | M | N | K | 0.90 | 0.76 | Y |
| 83 | 2016 | R255K | A | I | C | N | I | A | G | K | A | A | Y | F | S | N | D | C | V | I | E | T | 0.88 | 0.94 | Y |
| 85 | 2016 | K189T | A | I | R | N | I | S | A | K | A | A | N | Y | S | N | D | C | V | I | E | T | 0.68 | 0.64 | Y |
| 86 | 2016 | K189T | A | I | R | N | I | S | G | K | A | A | N | Y | S | N | D | C | V | M | N | K | 0.75 | 0.64 | Y |
| 87 | 2016 | WT | A | I | R | N | I | A | A | K | A | A | N | Y | S | N | D | C | V | M | N | K | 0.80 | 0.62 | Y |
| 88 | 2016 | K189T | A | I | R | N | I | A | G | K | A | A | N | F | S | N | D | C | V | M | N | K | 0.74 | 0.93 | Y |
| 90 | 2016 | WT | A | I | R | N | I | A | ND | K | A | A | Y | F | S | N | D | C | V | M | N | K | 0.75 | 0.72 | Y |
| 91 | 2016 | K189T/K | A | N/I | R | N | I | A | A | K | A | A | N | F | S | N | D | C | V | M | N | K | 0.88 | 0.72 | Y |
| 92 | 2016 | K189T | A | I | R | N | I | A | A | K | A | A | N | Y | S | N | D | C | V | M | N | K | 0.71 | 0.64 | Y |
| 93 | 2016 | K189T | A | I | R | N | I | A | A | K | A | A | N | Y/F | S | N | D | C | V | M | N | K | 0.79 | 0.69 | Y |
| 94 | 2016 | K189T | A | I | C | N | I | A | A | K | A | A | N | Y/F | S | N | D | C | V | M | N | K | 0.60 | 0.52 | Y |
| 95 | 2016 | WT | A | I | R | N | I | A | A | K | A | A | N | Y | S | N | D | C | V | M | N | K | 0.74 | 0.68 | Y |
| 96 | 2016 | WT | A | I | R | N | I | A | A | K | A | A | N | F | S | N | D | C | V | M | N | K | 0.86 | 0.83 | Y |
| 97 | 2016 | WT | A | N/I | R | N | I | S/A | A/G | K | A | A | N | Y | S | N | D | C | V | M | N | K/T | 0.75 | 0.93 | Y |
| 98 | 2016 | K189T | A | I | R | N | I | A | A | K | A | A | Y | F | S | N | D | C | V | M | N | K | 0.75 | 0.88 | Y |
| 99 | 2016 | WT | A | I | R | N | I | A | A | K | A | A | N | Y/F | S | N | D | C | V | M | N | K | 0.82 | 0.90 | Y |
| 100 | 2016 | K189T+N197D | A | I | R | N | I | A | A | K | A | A | N | Y | S | N | D | C | V | M | N | K | 0.70 | 0.93 | Y |
| 101 | 2016 | K189T | A | I | R | N | I | A | A | K | A | A | N | F | S | N | D | C | V | M | N | K | 0.89 | 1.01 | Y |
| 102 | 2016 | K189N | A | I | R | N | I | A | A | K | A | A | N | Y | S | N | D | C | V | M | N | K | 0.71 | 0.93 | Y |
| 103 | 2016 | K189T | A | I | R | N | I | A | A | K | A | A | N | Y/F | S | N | D | C | V | M | N | K | 1.06 | 0.96 | Y |
| 104 | 2016 | K189T | A | N | R | N | I | S | G | K | A | A | N | F | S | N | D | C | V | I | E | T | 0.97 | 0.70 | Y |
| 105 | 2016 | K189T/K | A | N/I | C/R | S/N | I | S/A | A/G | K | A | A | N/Y | Y | S | N | D | C | V | M | N | K | 0.50 | 1.06 | Y |
| 106 | 2016 | K189T | A | I | R | N | I | A | A | K | A | A | Y | F | S | N | D | C | V | M | N | K | 0.76 | 0.76 | Y |
| 107 | 2016 | WT | A | I | R | N | I | S | G | K | A | A | Y | F | S | N | D | C | V | M | N | K | 0.91 | 0.90 | Y |
| 108 | 2016 | K189T | A | I | R | N | I | S/A | G | K | A | A | N | Y/F | S | N | D | C | V | M/I | N/(A/G,A,A/T)* | K/T | 0.78 | 0.90 | Y |
| 109 | 2016 | K189T | A | N | R | N | I | A | A | K | A | A | N | ND | S | N | D | C | V | M | N | K | 0.99 | 0.87 | ND |
| 110 | 2016 | K189T | A | I | R | N | I | S/A | A | K | A | A | N | Y/F | S | N | D | C | V | M | N | K | 1.02 | 0.99 | Y |
| 111 | 2016 | Q633R | A | I | R | N | I | A | A | K | A | A | N | ND | S | N | D | C | V | M | N | K | ND | ND | Y |
| 112 | 2016 | ND | A | I | R | N | I | S | G | K | A | A | ND | ND | ND | ND | ND | C | V | I | E | T | ND | ND | ND |
| 113 | 2016 | K189T/K | A | I | R | N | I | A | A | K | A | A | N | Y/F | S | N | D | C | V | M | N | K | 0.95 | 0.94 | Y |
| 114 | 2016 | WT | A | N | R | N | I | A | A | K | A | A | N | Y | S | N | D | C | V | M | N | K | 0.76 | 0.76 | Y |
| 115 | 2016 | K189T | A | I | R | N | I | A | G | K | A | A | N | ND | S | N | D | C | V | M | N | K | 1.03 | ND | Y |
| 116 | 2016 | WT | A | I | R | N | I | S | G | K | A | A | N | F | S | N | D | C | V | M | N | K | 0.75 | 0.88 | Y |
| 117 | 2016 | WT | A | I | R | N | I | A | ND | K | A | A | N | Y | S | N | D | C | V | M | N | K | 0.89 | 0.91 | Y |
| 118 | 2016 | K189T | A | I | R | N | I | S | G | K | A | A | Y | F | S | N | D | C | V | M | N | K | 0.92 | 0.90 | Y |
| 119 | 2016 | ND | ND | ND | ND | ND | ND | ND | ND | ND | ND | ND | ND | ND | ND | ND | ND | C | V | M | N | K | ND | 0.86 | Y |
| 120 | 2016 | K189T | A | I | R | N | I | A | A | K | A | A | N | Y | S | N | D | C | V | M | N | K | 1.00 | 0.92 | Y |
| 121 | 2016 | K189T | A | I | R | N | I | A | ND | K | A | A | N | F | S | N | D | C | V | M | N | K | 1.06 | 0.91 | Y |
| 122 | 2016 | WT | A | I | R | N | I | A | G | K | A | A | N | ND | S | N | D | C | V | M | N | K | 0.98 | 1.09 | Y |
| 123 | 2016 | K189T | A | I | R | N | I | A | A | K | A | A | N | Y | S | N | D | C | V | M | N | K/T | 1.06 | 0.90 | Y |
| 124 | 2016 | WT | A | I | R | N | I | A | G | K | A | A | N | Y | S | N | D | C | V | M | N | K/T | 1.02 | 0.97 | Y |
| 125 | 2016 | WT | A | N | R | N | I | S | G | E | A | A | N | ND | S | N | D | C | V | M | N | K/T | 1.16 | 1.10 | Y |
| 126 | 2016 | K189T | A | N | R | N | I | A | A | K | A | A | N | F | S | N | D | C | V | M | N | K | 1.07 | 0.88 | Y |
| 127 | 2016 | K189T | A | I | R | N | I | A | A | K | A | A | N | F | S | N | D | C | V | M | N | K | 1.25 | 0.94 | Y |
| 128 | 2016 | K189T | A | I | R | N | I | A | A | K | A | A | N | F | S | N | D | C | V | M | N | K | 1.17 | 0.94 | Y |
| 129 | 2016 | K189T | A | N/I | R | N | I | A | A | K | A | A | N | F | S | N | D | C | V | M | N | K | 0.97 | 0.82 | Y |
| 130 | 2016 | WT | A | I | R | N | I | S | G | K | A | A | N | Y | S | N | D | C | V | M | N | K | 1.03 | 0.85 | ND |
| 131 | 2016 | K189T | A | I | R | N | I | A | A | K | A | A | N | F | S | N | D | C | V | M | N | K | 1.09 | 0.92 | Y |
| 132 | 2016 | K189T | A | N | R | N | I | S/A | G | K | A | A | N | F | S | N | D | C | V | M | N | K | 1.16 | 0.98 | Y |
| 133 | 2016 | WT | A | I | R | N | I | A | A | K | A | A | N | ND | S | N | D | C | V | M | N | K | 0.68 | 0.72 | Y |
| 134 | 2016 | K189T | A | I | R | N | I | S | G | K | A | A | N | F | S | N | D | C | V | M | N | K | 0.79 | 0.77 | Y |
| 135 | 2016 | K189T | A | I | R | N | I | S | A | K | A | A | N | Y | S | N | D | C | V | M | N | K | 0.94 | 0.76 | Y |
| 136 | 2016 | WT | A | I | R | N | I | A | A | K | A | A | N | F | S | N | D | C | V | I | E | T | 0.87 | 0.95 | Y |
| 137 | 2016 | K189T | A | I | R | N | I | A | A | K | A | A | N | Y | S | N | D | C | V | M | N | K | 0.78 | 0.90 | Y |
| 138 | 2016 | WT | A | I | R | N | I | A | A | K | A | A | N | Y | S | N | D | C | V | M | N | K | 0.88 | 0.91 | Y |
| 139 | 2016 | K189T | ND | ND | ND | ND | ND | A | ND | K | A | A | ND | ND | ND | ND | ND | C | V | M | N | K | ND | ND | Y |
| 140 | 2016 | K189T | A | I | R | N | I | A | G | K/E | A | A | N | F | S | N | D | C | V | M | N | K | 0.83 | 0.85 | Y |
| 141 | 2016 | K189T | A | I | R | N | I | S | G | K | A | A | N | Y | S | N | D | C | V | M | N | K | 0.73 | 0.78 | Y |
| 142 | 2016 | WT | A | I | R | N | I | S | G | K | A | A | N | Y | S | N | D | C | V | M | N | K | 0.90 | 0.90 | Y |
| 143 | 2016 | WT | A | N/I | R | N | I | S/A | A/G | K | A | A | N | F | S | N | D | C | V | M | N | K | 0.70 | 0.84 | Y |
| 144 | 2016 | K189N | A | I | R | N | I | A | A | K | A | A | N/Y | Y/F | S | N | D | C | V | M | N | K | 1.12 | 0.73 | ND |
| 145 | 2016 | K189T | A | I | R | N | I | A | G | K | G | S | N | F | S | N | D | C | V | I | E | T | 0.84 | 0.90 | Y |
| 146 | 2016 | WT | A | I | R | N | I | S | G | K | A | A | N | ND | S | N | D | C | V | M | N | K | 0.61 | 0.85 | ND |
| 147 | 2016 | K189T | A | I | R | N | I | A | G | K | A | A | N | Y | S | N | D | C | V | I | E | T | 0.93 | 0.93 | Y |
| 148 | 2016 | K189T | A | I | R | N | I | A | A/G | K | A | S | N | F | S | N | D | C | V | M/I | E | T | 1.19 | 0.71 | ND |
| 149 | 2016 | K189T | A | I | R | N | I | A | A | K | A | A | N | Y | S | N | D | C | V | M | N | K | 0.95 | 0.80 | Y |
| 150 | 2016 | WT | A | I | C | N | I | A | A | K | A | A | N | Y | S | N | D | C | V | M | N | K | 0.94 | 0.91 | ND |
| 151 | 2016 | K189T | A | I | R | N | I | A | A | K | A | A | N | Y | S | N | D | C | V | M/I | N/D | K/T | 0.70 | 0.88 | Y |
| 152 | 2016 | WT | A | I | R | N | I | A | A | K | A | A | N | F | S | N | D | C | V | M | N | K | 0.72 | 0.74 | Y |
| 154 | 2016 | K189T | A | N/I | R | N | I | A | A | K | A | A | N | F | S | N | D | C | V | M | N | K | 1.26 | 0.94 | Y |
| 155 | 2016 | WT | A | I | R | N | I | A | G | K | G | S | N | Y | S | N | D | C | V | M | N | K/T | 1.17 | 1.03 | Y |
| 156 | 2016 | K189T | A | I | R | N | I | A | A | K | A | A | N | Y | S | N | D | C | V | M | N | K | 0.78 | 0.78 | Y |
| 158 | 2016 | K189T | A | I | R | N | I | A | A | K | A | A | N | F | S | N | D | C | V | M | N | K | 1.15 | 0.92 | Y |
| 159 | 2016 | WT | A | I | R | N | I | A | A | K | A | A | N | F | S | N | D | C | V | M | N | K | 1.11 | 0.84 | Y |
| 161 | 2016 | K189T | A | N/I | R | N | I | A | A | K | A | A | N | F | S | N | D | C | V | M | N | K | 1.12 | 0.99 | Y |
| 162 | 2016 | R255K | A | I | R | N | I | S | G | K | A | A | N | F | S | N | D | C | V | M | N | K | 0.75 | 0.91 | Y |
| 163 | 2016 | WT | A | I | R | N | I | S/A | A | K | A | A | N | F | S | N | D | C | V | M | N | K | 1.03 | 1.09 | Y |
| 164 | 2016 | K189T | A | I | R | N | I | S | G | K | A | A | N | F | S | N | D | C | V | M | N | K | 1.06 | 0.86 | Y |
| 165 | 2016 | WT | A | N/I | R | N | I | A | A | K | A | A | N | Y | S | N | D | C | V | M | N | K | 1.14 | 0.92 | Y |
| 166 | 2016 | WT | A | I | R | N | I | A | A | K | A | A | N | Y | S | N | D | C | V | M | N | K | 1.22 | 0.86 | Y |
| 167 | 2016 | L258M | A | I | R | N | I | A | A/G | K | A | A | N | F | S | N | D | C | V | M | N | K | 0.72 | 0.91 | Y |
| 168 | 2016 | K189T | A | I | R | N | I | A | A | K | A | A | N | F | S | N | D | C | V | M | N | K | 0.92 | 0.57 | Y |
| 169 | 2016 | K189T/K | A | I | R | N | I | A | A | K | A | A | N | F | S | N | D | C | V | M | N | K | 1.16 | 0.86 | Y |
| 173 | 2016 | WT | A | I | R | N | I | A | ND | K | A | A | N | F | S | N | D | C | V | M | N | K | 0.76 | 0.82 | Y |
| 174 | 2016 | WT | A | I | R | N | I | S | G | K | A | A | N | F | S | N | D | C | V | M | N | K/T | 0.91 | 0.91 | Y |
| 175 | 2016 | K189T/K | A | I | R | N | I | A | A/G | K | A | A | N | F | S | N | D | C | V | M | N | K | 0.65 | 0.79 | Y |
| 176 | 2017 | A578S | A | I | R | N | I | A | A | K | A | A | N | F | S | N | D | C | V | M | N | K | 0.62 | 0.77 | Y |
| 177 | 2017 | K189T | A | I | R | N | I | A | A | K | A | A | N | Y | S | N | D | C | V | M | N | K | 0.87 | 0.95 | Y |
| 178 | 2017 | WT | A | I | R | N | I | A | A | K | A | A | N | F | S | N | D | C | V | M | N | K | 1.05 | 0.96 | Y |
| 179 | 2017 | WT | A | I | R | N | I | A | A | K | A | A | N | Y | S | N | D | C | V | M | N | K | 0.68 | 0.98 | Y |
| 180 | 2017 | WT | ND | ND | ND | ND | ND | S | G | K | A | A | N | Y | S | N | D | C | V | M | N | K | ND | ND | Y |
| 181 | 2017 | K189T | A | I | R | N | I | S | G | K | A | A | ND | ND | ND | ND | ND | C | V | M | N | K | 0.59 | 1.13 | Y |
| 182 | 2017 | K189T | A | I | R | N | I | A | A | K | A | A | N | F | S | N | D | C | V | M | N | K | 1.04 | 0.62 | Y |
| 183 | 2017 | K189T | ND | ND | ND | ND | ND | A | A/G | K | A | A | N | ND | S | N | D | C | V | M | N | K | 0.60 | 0.68 | ND |
| 184 | 2017 | K189N/K | A | I | R | N | I | A | A | K | A | A | N | F | S | N | D | C | V | M | N | K | 0.64 | 0.91 | Y |
| 185 | 2017 | WT | A | N | R | N | I | A | A | K | A | A | N | Y | S | N | D | C | V | M | N | K | 0.91 | 1.14 | Y |
| 186 | 2017 | K189T | A | I | R | N | I | A | A | K | A | A | N | Y | S | N | D | C | V | M | N | K | 0.56 | 0.75 | Y |
| 187 | 2017 | WT | A | I | R | N | I | S | G | K | A | A | N | Y | S | N | D | C | V | M | N | K | 0.71 | 0.86 | Y |
| 188 | 2017 | WT | A | N | R | N | I | A | A | K | A | A | N | F | S | N | D | C | V | M | N | K | 0.66 | 0.98 | Y |
| 189 | 2017 | K189T/K | A | I | R | N | I | S | G | K | A | A | N | F | S | N | D | C | V | M | N | K | 0.64 | 0.79 | Y |
| 190 | 2017 | WT | A | I | R | N | I | A | A | K | A | A | N | Y | S | N | D | C | V | M | N | K | 0.90 | 0.80 | Y |
| 191 | 2017 | K189T | A | I | R | N | I | S | G | K | A | A | N | Y | S | N | D | C | V | M | N | K | 1.28 | 0.81 | Y |
| 192 | 2017 | WT | A | I | R | N | I | A | A | K | A | A | N | Y | S | N | D | C | V | M | N | K | 0.83 | 0.93 | Y |
| 193 | 2017 | K189T | A | I | C/R | N | I | A | A | K | A | A | N | Y | S | N | D | C | V | M | N | K | 0.74 | 0.58 | Y |
| 194 | 2017 | WT | A | I | R | N | I | A | A | K | A | A | N | Y | S | N | D | C | V | M | N | K | 0.80 | 0.83 | Y |
| 195 | 2017 | WT | A | I | R | N | I | A | A | K | A | A | N | F | S | N | D | C | V | M | N | K | 0.77 | 0.76 | Y |
| 196 | 2017 | WT | A | I | R | N | I | A | A | K | A | A | N | F | S | N | D | C | V | M | N | K | 0.54 | 0.94 | Y |
| 197 | 2017 | K189T | A | I | R | N | I | A | A | K | A | A | N | F | S | N | D | C | V | M | N | K | 0.70 | 0.80 | Y |
| 198 | 2017 | K189T | A | I | R | N | I | A | A | K | A | A | N | F | S | N | D | C | V | M | N | K | 0.86 | 0.95 | Y |
| 199 | 2017 | WT | A | I | R | N | I | A | A | K | A | A | N | Y | S | N | D | C | V | M | N | K | 0.64 | 0.93 | Y |
| 200 | 2017 | S213G/S | A | I | R | N | I | A | A | K | A | A | N | F | S | N | D | C | V | M | N | K | 0.85 | 0.94 | Y |
| 201 | 2017 | WT | A | I | R | N | I | A | A | K | A | A | N | F | S | N | D | C | V | M | N | K | 0.71 | 0.97 | Y |
| 202 | 2017 | ND | ND | ND | ND | ND | ND | ND | ND | ND | ND | ND | ND | ND | ND | ND | ND | ND | ND | ND | ND | ND | ND | ND | Y |
| 203 | 2017 | WT | A | N | R | N | I | A | A | K | A | A | N | Y | S | N | D | C | V | M | N | K | 0.75 | 0.82 | Y |
| 204 | 2017 | R255K | A | I | R | N | I | A | A | K | A | A | N | Y | S | N | D | C | V | M | N | K | 0.77 | 0.95 | Y |
| 205 | 2017 | WT | A | I | R | N | I | C | A | K | A | A | N | Y | S | N | D | C | V | M | N | K | 0.92 | 0.84 | Y |
| 206 | 2017 | K189T | ND | ND | ND | ND | ND | A | A | K | A | A | ND | ND | ND | ND | ND | ND | ND | ND | ND | ND | ND | ND | ND |
| 207 | 2017 | K189T | A | I | R | N | I | S/A | A/G | K | A | A | N | Y | S | N | D | C | V | M/I | N/D | K | 0.88 | 1.05 | Y |
| 208 | 2017 | K189T | A | I | R | N | I | A | A/G | K | A | A | N | Y | S | N | D | C | V | M | N | K | 0.88 | 1.08 | Y |
| 209 | 2017 | R255K | A | I | R | N | I | A | G | K | A | A | N | Y | S | N | D | C | V | M | N | K | 1.23 | 0.89 | Y |
| 210 | 2017 | K189T | A | N | R | N | I | S | G | K | A | A | N | F | S | N | D | C | V | M | N | K | 1.29 | 1.11 | Y |
| 211 | 2017 | WT | A | I | R | N | I | A | A | K | A | A | N | F | S | N | D | ND | ND | ND | ND | ND | 1.28 | 1.50 | Y |
| 212 | 2017 | K189T/K | A | I | R | N | I | A | A/G | K | A | A | N | Y | S | N | D | C | V | M | N | K | 0.59 | 0.89 | Y |
| 213 | 2017 | K189T | A | I | R | N | I | S | G | K | A | A | N | F | S | N | D | C | V | M | N | K | 1.29 | 0.97 | Y |
| 214 | 2017 | WT | A | I | R | N | I | A | A | K | A | A | N | F | S | N | D | C | V | M | N | K | 0.93 | 0.95 | Y |
| 215 | 2017 | WT | A | I | R | N | I | A | G | K | G | S | N | F | S | N | D | C | V | M | N | K | 0.92 | 1.16 | Y |
| 216 | 2017 | K189T | A | I | R | N | I | A | A | K | A | A | N | Y | S | N | D | C | V | M | N | K | 0.97 | 1.01 | Y |
| 217 | 2017 | K189T | A | I | C | N | I | A | A | K | A | A | N | Y | S | N | D | C | V | M | N | K | 1.10 | 1.12 | Y |
| 218 | 2017 | K189T | A | I | C | N | I | A | A | K | A | A | N | Y | S | N | D | C | V | M | N | K | 1.02 | 1.04 | Y |
| 219 | 2017 | K189T | A | I | R | N | I | A | A | K | A | A | N | Y | S | N | D | C | V | M | N | K | 1.08 | 0.96 | Y |
| 220 | 2017 | WT | A | I | R | N | I | A | A | K | A | A | Y | F | S | N | D | C | V | M | N | K | 1.28 | 0.88 | Y |
| 221 | 2017 | K189T | A | I | R | N | I | A | A | K | A | A | N | F | S | N | D | C | V | M/I | D | T | 0.60 | 0.76 | Y |
| 222 | 2017 | K189T | A | I | R | N | I | S | G | K | A | A | N | Y | S | N | D | C | V | M | N | K | 1.06 | 0.99 | Y |
| 223 | 2017 | K189T | A | I | C/R | N | I | S/A | A/G | K | A | A | N | Y | S | N | D | C | V | M | N | K | 1.33 | 1.02 | Y |
| 224 | 2017 | K189T/K | A | N | R | N | I | A | G | K | G | S | N/Y | Y | S | N | D | C | V | M | N | K | 1.12 | 0.64 | Y |
| 225 | 2017 | K189T | A | I | R | N | I | A | G | K | A | A | N | F | S | N | D | C | V | M | N | K | 1.04 | 0.92 | Y |
| 226 | 2017 | WT | A | I | R | N | I | S | G | K | A | A | N | Y | S | N | D | C | V | M | N | K | 1.12 | 0.89 | Y |
| 227 | 2017 | WT | A | I | R | N | I | A | A | K | A | A | N | F | S | N | D | C | V | M | N | K | 1.28 | 0.85 | Y |
| 228 | 2017 | WT | A | N | R | N | I | A | A | K | A | A | N | Y | S | N | D | C | V | M | N | K | 0.86 | 0.90 | Y |
| 229 | 2017 | K189T/K | A | I | R | N | I | A | G | K | A | A | N | F | S | N | D | C | V | M | N | K | 0.70 | 0.93 | Y |
| 230 | 2017 | WT | ND | ND | ND | ND | ND | S | G | K | A | A | ND | ND | ND | ND | ND | ND | ND | ND | ND | ND | 1.17 | ND | ND |
| 231 | 2017 | K189T | A | I | R | N | I | A | A | K | A | A | N | Y | S | N | D | C | V | M | N | K | 0.75 | 0.95 | Y |
| 232 | 2017 | K189T/K, N197D/N | A | I | R | N | I | S/A | A/G | K | A | A | N | Y | S | N | D | C | V | M | N | K | 0.64 | 0.91 | Y |
| 234 | 2017 | K189T/K | A | I | R | N | I | A | A/G | K | A | A | N/Y | F | S | N | D | C | V | M | N | K | 0.64 | 0.50 | Y |
| 236 | 2017 | ND | A | N | R | N | I | S | G | K | A | A | N | F | S | N | D | C | V | M | N | K | 0.76 | 0.80 | Y |
| 238 | 2017 | K189T/K | A | I | R | N | I | A | A | K | A | A | N | Y | S | N | D | C | V | M | N | K | 0.80 | 0.93 | Y |
| 239 | 2017 | WT | A | I | R | N | I | S | G | K | A | A | Y | F | S | N | D | C | V | M | N | K | 0.61 | 0.86 | Y |
| 240 | 2017 | WT | A | I | R | N | I | A | A | K | A | A | N | Y | S | N | D | C | V | M | N | K | 0.66 | 0.80 | Y |
| 241 | 2017 | WT | A | I | R | N | I | A | A | K | A | A | N | F | S | N | D | C | V | M | N | K | 0.67 | 0.94 | Y |
| 242 | 2017 | ND | ND | ND | ND | ND | ND | A | A | K | A | A | N | Y | S | N | D | ND | ND | ND | ND | ND | 0.52 | 0.96 | ND |
| 243 | 2017 | K189T | A | I | R | N | I | A | G | K | G | S | N | F | S | N | D | C | V | M/I | N | K/T | 0.79 | 0.86 | Y |
| 244 | 2017 | WT | A | I | R | N | I | A | A | K | A | A | N | Y | S | N | D | C | V | M | N | K | 0.71 | 0.90 | Y |
| 245 | 2017 | WT | A | I | R | N | I | S/A | A/G | K | A | A | N | F | S | N | D | C | V | M | N | K | 1.24 | 0.92 | Y |
| 246 | 2017 | K189T | A | I | R | N | I | S | G | K | A | A | N | F | S | N | D | C | V | M | N | K | 0.83 | 1.02 | Y |
| 247 | 2017 | K189T/K | A | N | R | N | I | A | A | K | A | A | N | F | S | N | D | C | V | M | N | K | 0.81 | 0.96 | Y |
| 248 | 2017 | R255K | A | I | R | N | I | A | A | K | A | A | N | Y/F | S | N | D | C | V | M | N | K | 0.79 | 0.97 | Y |
| 249 | 2017 | ND | ND | ND | ND | ND | ND | ND | ND | ND | ND | ND | ND | ND | ND | ND | ND | ND | ND | ND | ND | ND | ND | ND | Y |
| 250 | 2017 | K189T | A | I | R | N | I | A | A | K | A | A | N | Y/F | S | N | D | C | V | M | N | K | 1.14 | 0.98 | Y |
| 251 | 2017 | K189T | A | I | R | N | I | A | A | K | A | A | N | Y/F | S | N | D | C | V | M | N | K | 0.75 | 0.64 | Y |
| 252 | 2017 | K189T | A | I | R | N | I | A | A | K | A | A | N | F | S | N | D | C | V | I | E | T | 0.99 | 1.08 | Y |
| 253 | 2017 | WT | A | I | R | N | I | S | G | K | A | A | N | F | S | N | D | C | V | I | E | T | 1.09 | 1.10 | Y |
| 254 | 2017 | K189T | A | I | R | N | I | A | A | K | A | A | N | Y | S | N | D | C | V | M | N | K | ND | 0.62 | Y |
| 255 | 2017 | K189T | A | I | C/R | N | I | S/A | A/G | K | A | A | N | Y | S | N | D | C | V | M/I | N/D | K/T | 1.01 | 0.80 | Y |
| 256 | 2017 | WT | A | I | R | N | I | S | G | K | A | A | N | Y | S | N | D | C | V | M | N | K | 1.19 | 0.99 | Y |
| 257 | 2017 | K189T | ND | ND | ND | ND | ND | A | A | K | A | A | ND | ND | ND | ND | ND | C | V | M | N | K | ND | ND | ND |
| 258 | 2017 | K189T | A | I | R | N | I | S | G | E | G | A | Y | F | S | N | D | C | V | M | N | K | 1.22 | 0.98 | Y |
| 259 | 2017 | WT | A | N/I | R | N | I | A | G | K | A | A | N | F | S | N | D | C | V | M | N | K | 1.06 | 0.94 | Y |
| 260 | 2017 | K189T | A | I | R | N | I | A | G | K | A | A | N | F | S | N | D | C | V | M | N | K | 0.98 | 1.02 | Y |
| 261 | 2017 | K189T, N197D/N | A | I | R | N | I | A | A | K | A | A | N | Y | S | N | D | C | V | M/I | N/D | K | 1.02 | 0.96 | Y |
| 262 | 2017 | ND | A | I | R | N | I | S/A | A/G | K | A | A | N | F | S | N | D | C | V | M | N | K | 1.15 | 1.01 | Y |
| 263 | 2017 | WT | A | N | C | S | I | A | A | K | A | A | N | F | S | N | D | C | V | M | N | K | 0.88 | 1.03 | Y |
| 264 | 2017 | WT | A | I | R | N | I | A | G | K | A | A | N | Y | S | N | D | C | V | M | N | K | 0.97 | 0.86 | Y |
| 265 | 2017 | ND | ND | ND | ND | ND | ND | ND | ND | ND | ND | ND | ND | ND | ND | ND | ND | C | V | M | N | K | ND | ND | ND |
| 266 | 2017 | ND | ND | ND | ND | ND | ND | ND | ND | ND | ND | ND | ND | ND | ND | ND | ND | C | V | M | N | K | ND | ND | Y |
| 267 | 2017 | K189T | A | I | C | N | I | A | A | K | A | A | N | F | S | N | D | C | V | M | N | K | 0.97 | 0.94 | Y |
| 268 | 2017 | WT | A | I | R | N | I | S | G | E | G | A | N | Y | S | N | D | C | V | M | N | K | 1.04 | 0.87 | Y |
| 269 | 2017 | WT | A | I | R | N | I | A | A | K | A | A | N | Y | S | N | D | C | V | M | N | K | 0.95 | 0.95 | Y |
| 270 | 2017 | WT | A | I | R | N | I | S | A | K | A | A | N | Y | S | N | D | C | V | M | N | K | 1.03 | 0.98 | Y |
| 272 | 2017 | WT | A | I | R | N | I | A | A | K | A | A | N | F | S | N | D | C | V | M | N | K | 0.89 | 1.03 | Y |
| 273 | 2017 | K189T | A | N/I | R | N | I | A | A | K | A | A | N | Y | S | N | D | C | V | M | N | K | 1.02 | 1.01 | Y |
| 274 | 2017 | WT | A | I | R | N | I | A | A | K | A | A | N | Y | S | N | D | C | V | M/I | N/D | K/T | 0.94 | 0.85 | Y |
| 275 | 2017 | K189T | A | I | R | N | I | A | G | K | A | A | Y | F | S | N | D | C | V | M/I | N/D | K/T | 0.80 | 0.71 | Y |
| 276 | 2017 | WT | A | I | R | N | I | A | A | K | A | A | N | F | S | N | D | C | V | M | N | K | 0.83 | 0.89 | Y |
| 277 | 2017 | K189T/K | A | I | R | N | I | A | G | K | A | A | N | F | S | N | D | C | V | M | N | K | 0.77 | 0.98 | Y |
| 278 | 2017 | WT | A | N | R | N | I | A | A | K | A | A | N | Y | S | N | D | C | V | M | N | K | 0.80 | 1.00 | Y |
| 279 | 2017 | K189T | A | N | R | N | I | S | G | E | G | A | ND | ND | ND | ND | ND | C | V | M | N | K | 1.00 | 0.95 | Y |
| 280 | 2017 | K189T/K | A | I | R | N | I | S/A | A | K | A | A | N | F | S | N | D | C | V | M | N | K | ND | 0.52 | Y |
| 281 | 2017 | K189T | A | I | R | N | I | A | A | K | A | A | ND | ND | ND | ND | ND | C | V | I | D | K | 1.23 | 0.63 | Y |
| 283 | 2017 | K189T | A | N | C | S | I | A | A | K | A | A | N | Y | S | N | D | C | V | M | N | K | 1.07 | 1.03 | Y |
| 284 | 2017 | K189T | A | I | R | N | I | A | G | K | G | S | N | Y | S | N | D | C | V | M/I | N/D | K/T | 0.75 | 1.04 | Y |
| 285 | 2017 | K189T/K | A | N | C | S | I | S/A | A/G | K | A | A | N | Y | S | N | D | C | V | M | N | K | 1.09 | 0.93 | Y |
| 287 | 2017 | WT | A | I | R | N | I | A | G | K | A | A | N | Y/F | S | N | D | C | V | M | N | K | 0.96 | 0.88 | Y |
| 288 | 2017 | K189T | A | I | R | N | I | A | A | K | A | A | N | F | S | N | D | C | V | M | N | K | 0.88 | 0.96 | Y |
| 289 | 2017 | K189T/K | A | I | R | N | I | A | A | K | A | A | N | F | S | N | D | C | V | M | N | K | 0.78 | 1.02 | Y |
| 290 | 2017 | WT | A | I | R | N | I | A | A | K | A | A | N | F | S | N | D | C | V | M/I | N/D | K/T | 0.77 | 0.94 | Y |
| 291 | 2017 | K189T | A | I | R | N | I | S | G | K | A/G | A | N | F | S | N | D | C | V | M | N | K | 0.76 | 0.91 | Y |
| 292 | 2017 | K189T | A | I | R | N | I | A | A | K | A | A | N | Y/F | S | N | D | C | V | M | N | K | 0.96 | 0.97 | Y |
| 293 | 2017 | K189T | A | I | R | N | I | A | A | K | A | A | N | F | S | N | D | C | V | M/I | N/D | K/T | 0.84 | 0.94 | Y |
| 295 | 2017 | K189T/K | A | I | R | N | I | S/A | A/G | K | A | A | N | F | S | N | D | C | V | M | N | K | 0.68 | 1.04 | Y |
| 296 | 2017 | ND | ND | ND | ND | ND | ND | ND | ND | ND | ND | ND | ND | ND | ND | ND | ND | ND | ND | ND | ND | ND | ND | ND | Y |
| 297 | 2017 | K189T/K | A | I | R | N | I | A | A | K | A | A | N | Y/F | S | N | D | C | V | M/I | N/D | K/T | 1.18 | 0.93 | Y |
| 298 | 2017 | ND | A | I | R | N | I | S/A | A/G | K | A | A | N | F | S | N | D | C | V | M | N | K | 1.24 | 0.76 | Y |
| 299 | 2017 | WT | A | I | R | N | I | A | A | K | A | A | N | F | S | N | D | C | V | M | N | K | 1.24 | 1.05 | Y |
| 300 | 2017 | ND | ND | ND | ND | ND | ND | S | G | K | A | A | N | F | S | N | D | C | V | M | N | K | 0.78 | 0.60 | Y |
| 301 | 2017 | K189T | A | N | R | N | I | A | A | K | A | A | N | Y | S | N | D | C | V | M | N | K | 1.09 | 0.97 | Y |
| 302 | 2017 | WT | A | I | R | N | I | S | G | K | A | A | N | Y/F | S | N | D | C | V | M | N | K | 1.09 | 0.80 | Y |
| 303 | 2017 | WT | A | I | R | N | I | A | A | K | A | A | N | F | S | N | D | C | V | M | N | K | 1.23 | 0.89 | Y |
| 305 | 2017 | K189T | A | I | R | N | I | A | A | K | A | A | N | Y | S | N | D | C | V | M | N | K | 1.03 | 1.00 | Y |
| 306 | 2017 | WT | A | I | R | N | I | S/A | A/G | K | A/G | A | N | F | S | N | D | C | V | M | N | K | 1.00 | 0.93 | Y |
| 307 | 2017 | WT | A | I | R | N | I | S | G | K | A | A | N | Y | S | N | D | C | V | I | E | T | 1.20 | 0.96 | Y |
| 308 | 2017 | K189T | A | I | R | N | I | A | A | K | A | A | N | Y | S | N | D | C | V | M | N | K | 1.09 | 1.00 | Y |
| 309 | 2017 | K189T/K | A | I | R | N | I | A | A/G | K | A | A | N | F | S | N | D | C | V | M | N | K | 1.21 | 0.90 | Y |
| 310 | 2017 | WT | A | I | R | N | I | S | G | K | A | A | N | F | S | N | D | C | V | M | N | K | 1.01 | 1.01 | Y |
| 311 | 2017 | K189T | A | I | R | N | I | A | A | K | A | A | N | F | S | N | D | C | V | M/I | N/D | K/T | 0.93 | 0.92 | Y |
| 312 | 2017 | ND | ND | ND | ND | ND | ND | ND | ND | ND | ND | ND | ND | ND | ND | ND | ND | ND | ND | ND | ND | ND | ND | ND | Y |
| 314 | 2017 | K189T | A | I | R | N | I | S | G | E | G | A | N | F | S | N | D | C | V | M | N | K | 0.62 | 0.97 | Y |
| 315 | 2017 | WT | A | I | R | N | I | A | A | K | A | A | N | F | S | N | D | C | V | M | N | K | 0.66 | 1.00 | Y |
| 316 | 2017 | K189T | A | I | R | N | I | S | G | K | A | A | N | Y | S | N | D | C | V | M | N | K | 0.68 | 0.87 | Y |
| 317 | 2017 | K189T | A | I | R | N | I | A | G | K | A | A | N | F | S | N | D | C | V | M | N | K | 0.81 | 1.00 | Y |
| 318 | 2017 | WT | A | I | R | N | I | S | G | K | A | A | N | F | S | N | D | C | V | M | N/D | K/T | 1.06 | 1.05 | Y |
| 319 | 2017 | WT | A | I | C/R | N | I | S/A | G | K | A | A | N | Y/F | S | N | D | C | V | M/I | N/D | K/T | 0.58 | 0.94 | Y |
| 320 | 2017 | K189T | A | N | R | N | I | A | A | K | A | A | N | F | S | N | D | C | V | M | N | K | 0.76 | 0.83 | Y |
| 321 | 2017 | WT | A | I | R | N | I | S | G | K | A | A | N | Y | S | N | D | C | V | M | N | K | 0.93 | 0.70 | Y |
| 322 | 2017 | K189T | A | I | R | N | I | A | A | K | A | A | N | F | S | N | D | C | V | M/I | N/D | K/T | 0.60 | 0.75 | Y |
| 323 | 2017 | WT | A | I | R | N | I | A | A/G | K | A | A | N | F | S | N | D | C | V | M | N | K | 0.89 | 0.88 | Y |
| 324 | 2017 | K189T | A | N | R | N | I | S | G | K | A | A | N | F | S | N | D | C | V | M | N | K | 0.80 | 0.86 | Y |
| 325 | 2017 | WT | A | I | R | N | I | A | A | K | A | A | N | Y | S | N | D | C | V | M | N | K | 0.81 | 0.89 | Y |
| 326 | 2017 | WT | A | N | C | S | I | A | G | K | G | S | N | F | S | N | D | C | V | M | N | K | 0.62 | 0.76 | Y |
| 327 | 2017 | K189T | A | I | R | N | I | A | A/G | K | A | A | N | F | S | N | D | ND | ND | ND | ND | ND | 0.77 | 0.95 | Y |
| 328 | 2017 | WT | A | I | R | N | I | S | G | K | A | A | N | Y | S | N | D | C | V | M | N | K | 0.69 | 0.83 | Y |
| 329 | 2017 | K189T | A | N/I | R | N | I | A | A | K | A | A | N | Y | S | N | D | C | V | M | N | K | 0.74 | 0.68 | Y |
| 330 | 2017 | WT | A | I | R | N | I | A | A | K | A | A | N | Y/F | S | N | D | C | V | M | N | K | 0.86 | 0.95 | Y |
| 331 | 2017 | WT | A | N | R | N | I | A | A | K | A | A | N | Y | S | N | D | C | V | I | E | T | 0.81 | 0.82 | Y |
| 332 | 2017 | K189T | A | I | R | N | I | A | A | K | A | A | N | Y | S | N | D | C | V | M/I | N/D | K/T | 0.95 | 0.88 | Y |
| 333 | 2017 | WT | A | I | C | N | I | A | A | K | A | A | N | F | S | N | D | C | V | M | N/D | K/T | 0.81 | 0.93 | Y |
| 334 | 2017 | WT | A | I | R | N | I | A | A | K | A | A | N | F | S | N | D | C | V | M | N | K | 0.97 | 0.90 | Y |
| 335 | 2017 | K189T | A | N/I | R | N | I | A | A | K | A | A | N | Y/F | S | N | D | C | V | M | N | K | 0.89 | 0.86 | Y |
| 336 | 2017 | K189T | A | I | R | N | I | S | G | K | A | A | N | Y | S | N | D | C | V | M | N | K | 0.90 | 0.82 | Y |
| 337 | 2017 | R255K | A | I | R | N | I | ND | ND | ND | ND | ND | Y | Y | S | N | D | C | V | I | E | T | ND | ND | Y |
| 338 | 2017 | K189T | A | I | R | N | I | ND | ND | ND | ND | ND | N | F | S | N | D | C | V | M | N | K | 0.76 | ND | ND |
| 339 | 2017 | R255K | A | I | R | N | I | A | A | K | A | A | N | F | S | N | D | C | V | M | N | K | 1.15 | 0.74 | Y |
| 340 | 2017 | WT | A | I | R | N | I | A | A | K | A | A | N | Y/F | S | N | D | C | V | M | N | K | 1.16 | 1.01 | Y |
| 341 | 2017 | K189T | A | I | R | N | I | S | G | K | A | A | N | F | S | N | D | C | V | M | N | K | 1.05 | 0.88 | ND |
| 342 | 2017 | K189T | A | I | R | N | I | A | A | K | A | A | N | F | S | N | D | C | V | M | N | K | 1.20 | 1.20 | Y |
| 344 | 2017 | N195D/N | A | N | R | N | I | A | A | K | A | A | N | Y | S | N | D | C | V | M | N | K | 0.80 | 0.97 | Y |
| 346 | 2017 | K189T | A | N/I | R | N | I | A | A | K | A | A | N | Y | S | N | D | C | V | M | N | K | 1.13 | 0.94 | Y |
| 347 | 2017 | K189T | A | I | C/R | N | I | A | A | K | A | A | N | F | S | N | D | C | V | M | N | K | ND | 1.02 | ND |
| 348 | 2017 | WT | A | N | R | N | I | S | A | K | A | A | N | F | S | N | D | C | V | M | N | K | 0.78 | 0.99 | Y |
| 349 | 2017 | K189T | A | I | R | N | I | A | A | K | A | A | N | F | S | N | D | C | V | M | N | K | ND | 0.93 | Y |
| 350 | 2017 | K189T | A | N | R | N | I | A | A | K | A | A | N | F | S | N | D | C | V | M | N | K | 1.08 | 0.99 | Y |
| 351 | 2017 | K189T | A | I | R | N | I | A | A | K | A | A | N | Y | S | N | D | C | V | I | E | T | ND | 0.91 | Y |
| 352 | 2017 | K189T | A | I | R | N | I | S | G | E | G | A | N | F | S | N | D | C | V | M | N | K | 1.24 | 0.97 | Y |
| 353 | 2017 | WT | A | I | R | N | I | A | A | K | A | A | N | F | S | N | D | C | V | M | N | K | 0.60 | 0.97 | Y |
| 354 | 2017 | K189T | A | I | R | N | I | A | A | K | A | A | N | Y | S | N | D | C | V | M | N | K | 0.99 | 1.07 | Y |
| 355 | 2017 | K189T | A | I | R | N | I | S | G | E | A | A | N | F | S | N | D | C | V | M | N | K | 0.86 | 1.06 | Y |
| 356 | 2017 | K189T | A | I | R | N | I | A | A | K | A | A | N | F | S | N | D | C | V | M | N | K | 0.62 | 1.14 | Y |
| 357 | 2017 | WT | A | I | R | N | I | A | A | K | A | A | N | Y | S | N | D | C | V | M | N | K | 0.72 | 1.00 | Y |
| 358 | 2017 | ND | ND | ND | ND | ND | ND | ND | ND | ND | ND | ND | ND | ND | ND | ND | ND | ND | ND | ND | ND | ND | ND | ND | Y |
| 359 | 2017 | K189T | ND | ND | ND | ND | ND | S | G | E | G | A | ND | ND | ND | ND | ND | ND | ND | ND | ND | ND | ND | 1.23 | Y |
| 360 | 2017 | K189T | A | I | R | N | I | A | A | K | A | A | N | F | S | N | D | C | V | M | N | K | 0.59 | 0.86 | Y |
| 362 | 2017 | ND | A | I | C | N | I | A | A | K | A | A | N | Y | S | N | D | ND | ND | ND | ND | ND | 0.74 | 1.00 | Y |
| 363 | 2017 | K189T | A | I | R | N | I | A | A | K | A | A | N | Y | S | N | D | C | V | M | N | K | 0.66 | 0.69 | Y |
| 364 | 2017 | ND | ND | ND | ND | ND | ND | A | A | K | A | A | ND | ND | ND | ND | ND | ND | ND | ND | ND | ND | ND | ND | ND |
| 365 | 2017 | ND | A | I | R | N | I | S | G | K | A | A | N | Y | S | N | D | C | V | M | N | K | 0.69 | ND | Y |
| 366 | 2017 | K189T/K | A | I | R | N | I | S | A/G | K | A | A | N | Y | S | N | D | C | V | M | N/D | K/T | 0.57 | 0.72 | Y |
| 367 | 2017 | WT | A | I | R | N | I | S | A | K/E | G | A | N | F | S | N | D | C | V | M | N | K | 0.61 | 1.00 | Y |
| 368 | 2017 | K189T | A | I | R | N | I | A | A | K | A | A | N | Y | S | N | D | C | V | M | N | K | 0.68 | 0.95 | Y |
| 369 | 2017 | WT | A | I | R | N | I | A | G | K | G | S | N | Y | S | N | D | C | V | M | N | K | 0.78 | 0.96 | Y |
| 370 | 2017 | K189T | A | I | R | N | I | A | A | K | A | A | N | Y | S | N | D | C | V | M | N | K | ND | ND | Y |
| 371 | 2017 | K189T/K | A | I | R | N | I | A | G | K | G | S | N | F | S | N | D | C | V | M | N | K | 0.67 | 0.92 | Y |
| 372 | 2017 | WT | A | I | R | N | I | S | G | K | A | A | N | F | S | N | D | C | V | M | N | K | 0.79 | 1.00 | Y |
| 373 | 2017 | K189T | A | I | R | N | I | S | G | E | G | A | N | F | S | N | D | C | V | M | N | K | 0.88 | 0.89 | Y |
| 374 | 2017 | K189T/K | A | I | C/R | N | I | A | A | K | A | A | N | F | S | N | D | C | V | M | N | K | 0.95 | 0.95 | Y |
| 375 | 2017 | K189T | A | I | R | N | I | A | G | K | A | A | N | F | S | N | D | C | V | M | N | K | 0.92 | 0.77 | Y |
| 376 | 2017 | K189T, V636A/V | ND | ND | ND | ND | ND | A | A | K | A | A | N | F | S | N | D | C | V | M | N | K | 0.77 | 0.57 | Y |
| 377 | 2017 | WT | A | I | R | N | I | S | G | K | A | A | N | Y | S | N | D | C | V | M | N | K | 0.93 | 0.81 | Y |
| 378 | 2017 | K189T | A | I | R | N | I | S | G | K | A | A | N | Y | S | N | D | C | V | M | N | K | 1.08 | 1.09 | Y |
| 379 | 2017 | WT | A | I | R | N | I | A | A | K | A | A | ND | ND | ND | ND | ND | C | V | M | N | K | 0.91 | 1.03 | Y |
| 381 | 2017 | WT | A | I | R | N | I | A | A | K | A | A | N | F | S | N | D | C | V | M | N | K | 0.92 | 1.16 | Y |
| 382 | 2017 | K189N | A | N | R | N | I | A | A | K | A | A | N | Y | S | N | D | C | V | M | N | K | 1.23 | 1.27 | Y |
| 383 | 2017 | ND | A | N | R | N | I | A | A | K | A | A | N/Y | Y | S | N | D | ND | ND | ND | ND | ND | ND | ND | Y |
| 384 | 2017 | WT | A | I | R | N | I | A | A | K | A | A | N | Y | S | N | D | C | V | M | N | K | 1.09 | 0.85 | Y |
| 385 | 2017 | K189T, W660C | A | I | R | N | I | S | G | K | A | A | N | Y | S | N | D | C | V | M | N | K | ND | ND | Y |
| 386 | 2017 | WT | A | I | R | N | I | A | A | K | A | A | ND | ND | ND | ND | ND | C | V | M | N | K | ND | ND | Y |
| 387 | 2017 | WT | A | I | R | N | I | A | A/G | K | A | A | N | F | S | N | D | C | V | M | N | K | ND | 1.10 | Y |
| 388 | 2017 | N195K/N | A | I | R | N | I | A | A | K | A | A | N | F | S | N | D | C | V | M | N | K | 1.03 | 1.08 | Y |
| 389 | 2017 | K189T | A | I | R | N | I | S | G | K | A | A | N | Y | S | N | D | C | V | I | E | T | 1.21 | 0.65 | Y |
| 390 | 2017 | K189T | A | I | R | N | I | S | G | E | A | A | N | Y | S | N | D | C | V | M | N | K | 1.04 | 1.09 | Y |
| 391 | 2017 | WT | A | I | R | N | I | A | A | K | A | A | Y | F | S | N | D | C | V | M | N | K | 1.08 | 1.05 | Y |
| 392 | 2017 | K189N/K | ND | ND | ND | ND | ND | ND | ND | ND | ND | ND | ND | ND | ND | ND | ND | C | V | M | N | K | 1.27 | 0.77 | ND |
| 393 | 2017 | K189T | A | I | C | N | I | S | G | E | A | A | N | F | S | N | D | C | V | M/I | N/D | T | 0.65 | 1.11 | Y |
| 394 | 2018 | WT | A | I | R | N | I | A | G | K | G | S | N | Y | S | N | D | C | V | I | E | T | 0.78 | 1.03 | Y |
| 395 | 2018 | K189T | A | I | R | N | I | ND | ND | ND | ND | ND | N | Y | S | N | D | C | V | M | N | K | ND | ND | Y |
| 397 | 2018 | ND | ND | ND | ND | ND | ND | ND | ND | ND | ND | ND | ND | ND | ND | ND | ND | C | V | M | N | K | ND | ND | Y |
| 398 | 2018 | WT | A | I | R | N | I | A | A/G | K | A | A | N | F | S | N | D | C | V | M | N | K | 1.06 | 0.62 | Y |
